# Free Brick1 Is a Trimeric Precursor in the Assembly of a Functional Wave Complex

**DOI:** 10.1371/journal.pone.0002462

**Published:** 2008-06-18

**Authors:** Emmanuel Derivery, Jenny Fink, Davy Martin, Anne Houdusse, Matthieu Piel, Theresia E. Stradal, Daniel Louvard, Alexis Gautreau

**Affiliations:** 1 Institut Curie, Centre de Recherche, “Morphogenesis and Cell Signaling” laboratory, Paris, France; 2 Institut Curie, Centre de Recherche, “Systems Cell Biology of Cell Polarity and Cell Division” laboratory, Paris, France; 3 Institut Curie, Centre de Recherche, “Structural Motility” laboratory, Paris, France; 4 CNRS UMR144, Paris, France; 5 HZI-Helmholz Center for Infection Research, Braunschweig, Germany; Duke University Medical Centre, United States of America

## Abstract

**Background:**

The Wave complex activates the Arp2/3 complex, inducing actin polymerization in lamellipodia and membrane ruffles. The Wave complex is composed of five subunits, the smallest of which, Brick1/Hspc300 (Brk1), is the least characterized. We previously reported that, unlike the other subunits, Brk1 also exists as a free form.

**Principal Findings:**

Here we report that this free form of Brk1 is composed of homotrimers. Using a novel assay in which purified free Brk1 is electroporated into HeLa cells, we were able to follow its biochemical fate in cells and to show that free Brk1 becomes incorporated into the Wave complex. Importantly, incorporation of free Brk1 into the Wave complex was blocked upon inhibition of protein synthesis and incorporated Brk1 was found to associate preferentially with neosynthesized subunits. Brk1 depleted HeLa cells were found to bleb, as were Nap1, Wave2 or ARPC2 depleted cells, suggesting that this blebbing phenotype of Brk1 depleted cells is due to an impairment of the Wave complex function rather than a specific function of free Brk1. Blebs of Brk1 depleted cells were emitted at sites where lamellipodia and membrane ruffles were normally emitted. In Brk1 depleted cells, the electroporation of free Brk1 was sufficient to restore Wave complex assembly and to rescue the blebbing phenotype.

**Conclusion:**

Together these results establish that the free form of Brk1 is an essential precursor in the assembly of a functional Wave complex.

## Introduction

Cell morphogenesis relies on the shape of the plasma membrane, which is controlled by the cortical actin cytoskeleton. Actin polymerization provides the force projecting the plasma membrane [1]. The Arp2/3 complex plays an essential role in actin nucleation in these plasma membrane projections [2], [3]. The Arp2/3 complex is composed of seven subunits. It requires both a mother filament and an activator in order to initiate the nucleation of a daughter filament through a conformational rearrangement of its two actin-related proteins [1]. The Wave proteins (also known as Scar) are direct activators of the Arp2/3 complex *in vitro* [4]. They are required for plasma membrane projections in diverse systems such as lamellipodia formation in migrating animal cells [5], [6], dendritic spine morphology in neurons [7] or trichome morphogenesis in plant cells [8], [9].

Wave proteins are subunits of a multiprotein complex [10], [11]. Although the molecular mechanism by which the Wave protein is regulated is still a matter of debate, it has become generally accepted that this multiprotein complex containing Wave is the major regulator of Wave activity [12]. Indeed, genetic inactivation of subunits of the Wave complex led to phenotypes similar to the inactivation of the Wave protein itself in a variety of plant and animal systems [9], [13], [14], [15], [16], [17]. The stability of the Wave complex makes it amenable to purification. The major Wave complex expressed by HeLa cells is composed of Sra1, Nap1, Wave2, Abi1 and Brk1 [11], yet in other cell lines or in different tissues, several homologous subunits encoded by paralogous genes are expressed, adding a combinatorial complexity to the Wave complexes [10], [15]. The only subunit for which no paralogous gene has been identified in any plant or animal species is the small subunit Brk1 (around 80 amino-acids, depending on the species). Brk1 is also the most highly conserved subunit (table S1). In this report, we will use the name Brick1, abbreviated to Brk1, for all Brk1 proteins from plants as well as from animals, because Hspc300 refers to a particular human cDNA (Genbank Accession number AF161418) in which the stop codon is mutated resulting in an artificially elongated protein. Moreover, Brk1 was first identified in a genetic screen for morphogenetic mutants of maize [18]. Brk1 is the only subunit for which a major pool is not associated with the Wave complex [11]. Here we report that this free pool of Brk1 is composed of an homotrimer and that the Brk1 homotrimer is a critical precursor for the assembly of the Wave complex.

## Methods

### Cells, transfection and protein electroporation

293T cells were transfected with plasmids using the standard calcium phosphate procedure. HeLa cells were transfected with 20 nM of Smartpool On target plus siRNA (Dharmacon) using Lipofectamine RNAiMAX (Invitrogen) in regular tissue culture dishes. Sequences of Brk1 siRNAs are given in the supplementary material. To electroporate HeLa cells with purified Brk1 proteins, cells were resuspended at 25×10^6^ cells/ml in DMEM/10% FCS containing 15 mM Hepes pH 7.5. 200 µl of cells were mixed to 50 µl of purified protein in PBS (12 µM, roughly 10 µg for regular assays, and up to 40 µg for the rescue experiment). Cells were pulsed at 240 V and 950 µF in a 0.4 cm wide cuvette in a BioRad GenepulserII. Cells were then quickly resuspended in the same medium, washed once and incubated in a 10 cm dish previously coated with 20 µg/ml Collagen type I (Becton-Dickinson). Cycloheximide (Sigma) was used at 1 mM.

### Imaging and Micropatterns

For scoring the blebbing phenotype, siRNA treated cells were trypsinized and seeded on regular plastic tissue culture dishes after 48 hrs of treatment. They were then observed live at 72 hrs by phase contrast optics and counted as blebbing or not. Statistics on blebbing cells were performed using the Sigmastat software (Systat), and all data analyzed passed the required tests of normality and equal variance.

For regular time lapse microscopy without micropatterns, siRNA treated cells were trypsinized and seeded on uncoated glass coverslips after 48 hrs of treatment. They were then imaged live at 72 hrs with an inverted Leica DMIRBE microscope equipped with a heating stage, a 63× oil-immersion objective and a CoolSnapEz camera (Photometrics).

For micropatterning, poly-dimethyl siloxane stamps (Sylgard 184 Kit, Dow Corning) were made as previously described [19]. A stamp was inked with a fibronectin/collagen solution (50 mg/ml each in H_2_O; Sigma-Aldrich). 10% of the fibronectin was labelled with Cy3 (Amersham Biosciences). After 45 min incubation in the dark, the stamp was dried, immediately placed in contact with a glass coverslip (Marienfeld) and slightly pressed with tweezers for several seconds. After 5 min, the stamp was removed, the coverslip was washed once with water and incubated in 10 mM Hepes, pH 7.4 containing 0.1 mg/ml PLL(20)-g[3.5]-PEG(2) (SurfaceSolutionS) for 1 hour. The coverslip was then rinced once with PBS and used for experiments. SiRNA treated cells used for micropattern experiments were trypsinized and seeded on regular plastic tissue culture dishes after 48 hrs of treatment. After 72 hrs, they were detached and dissociated with PBS containing 0.02% EDTA at 37°C for 15 min. After washing in DMEM containing 1% FCS, cells were seeded on micropatterned surfaces at a density of 1.5×10^4^ cells per cm^2^. After 1 hr, floating cells were removed. After a total of 4 hrs of spreading on micropatterns, cells were imaged with a 40× oil-immersion objective on an inverted IX71 Olympus microscope or with a 40× dry objective on a Nikon TE2000-E. Both were equipped with a Coolsnap HQ Roper camera (Photometrics), a temperature control chamber from Life Imaging Services (The Cube) combined with a CO_2_/hygrometry control from Oko Lab and a motorized stage (Märzhauser) to perform multifield acquisition.

All time lapse microscopy was done in Hepes-supplemented DMEM medium without phenol red. All microscopes were operated with Metamorph 7 (Universal Imaging). Handling of movies was performed using Metamorph, ImageJ and Adobe Premiere softwares.

### Antibodies

A monoclonal antibody (mAb) against Brk1 was obtained after immunization of mice with purified human Brk1 obtained from *E.coli*. The hybridoma supernatants were screened in two sandwich ELISAs using either Wave2 antibodies or Brk1 rabbit polyclonal antibodies. The IgG2b mAb 231H9 scored positive in both assays. Its characterization is displayed in Fig. S1. Wave2, Brk1, Abi1, Sra1 rabbit polyclonal antibodies were previously described [11]. Flag M2 mAb, tubulin α mAb, Actin mAb, and HA pAb were from Sigma. Arp3 and ARPC2 pAb were from Upstate biotechnology.

### Plasmids

All Brk1 constructs used for *in vitro* translation were derived from pCS2. Drosophila Brk1 (CG30173) was cloned from genomic DNA after fusing the two exons by PCR-mediated recombination. *Zea mays* Brk1 cDNA was provided by Laurie Smith (UCSD, CA). *Arabidopsis thaliana* Brk1 (AF370530; pda00573) was provided by the RIKEN Genomic Sciences Center (Japan). All tags were N-terminal and encoded the following peptides:

Flag, MDYKDDDDK;
PC, MEDQVDPRLIDGKEFDGRP;
SBP, MDEKTTGWRGGHVVEGLAGELEQLRARLEHHPQGQREPGSGHHHHHHEFDGRP amplified from pTAG2K [20];
HA, MYPYDVPDYAYPYDVPDYAYPYDVPDYAGRPPPPPPRP.

### In vitro translation, immunoprecipitations and SDS-PAGE

*In vitro* translated products were obtained using TnT coupled systems using either rabbit reticulocyte lysate or wheat germ extract for plant proteins (both from Promega) together with [^35^S] labeled methionine (Amersham Biosciences, GE Healthcare). pCS2 plasmids were transcribed with SP6. IVT products were diluted 10-fold in PBS supplemented with BSA 0.1%, Protease Inhibitor Cocktail (1∶1000, Sigma) and AEBSF 1 mM (Interchim), and immunoprecipitated. For metabolic labeling, cells were rinsed in DMEM lacking methionine and cysteine (Gibco, Invitrogen) supplemented with 250 µCi/ml redivue cell labeling Pro-Mix containing [^35^S] methionine and cysteine (Amersham biosciences, GE Healthcare) and 10% fetal calf serum dialyzed against PBS. Cells were lysed in RIPA (50 mM Hepes, 150 mM NaCl, 5 mM EDTA, 1% NP-40, 0,5% DOC, 0.1% SDS; pH 7.7) supplemented with Protease Inhibitor Cocktail (1∶1000, Sigma) and AEBSF 1 mM (Interchim). For PC immunoprecipitations, buffers contained 1 mM Ca^2+^ (and no EDTA), and anti-Protein C affinity beads (Roche) were eluted by the addition of 5 mM EDTA. SBP tagged material was precipitated with ultralink immobilized streptavidin plus beads (Pierce) and eluted by 100 µM free biotin (Fluka). Antibodies were precipitated with Ultralink immobilized protein G plus beads (Pierce). After rotating the bead suspension for 2 hours at 4°C, beads were washed four times in the lysis buffer, before specific elution or boiling in the SDS PAGE loading buffer. SDS-PAGE was performed using NuPAGE gels (Invitrogen): 4–12% Bis-Tris gels to detect Brk1 (or 12% Bis-Tris gels for the 4 tag experiment) using MES running buffer, and 3–8% Tris-Acetate gels. Gels were transferred on nitrocellulose membranes according to the manufacturer's instructions. Western blots were revealed with alkaline-phosphatase coupled antibodies and NBT/BCIP (Promega) or alternatively with HRP coupled antibodies, ECL and quantification with a Fuji LAS-3000 (Fujifilm). Blots were quantified using ImageJ software.

Colloidal Coomassie (Invitrogen) was used for total protein staining of the gel. For autoradiography, gels were fixed for 20 min, rinsed in water, incubated for 20 min in 1 M sodium salicylate, dried and exposed to films or phosphorimager for quantification (STORM 860, ImageQuant software, Molecular Dynamics).

### Protein purification and cross-linking

Human Brk1 was purified from *E.coli* using a modified pACYC vector encoding a fusion protein displaying 6xHis, GST, a TEV protease cleavage site then hBrk1. Briefly, the fusion protein was first purified on Glutathione Sepharose, eluted off the beads, and cleaved with TEV protease. The resulting Brk1 protein had the extra amino-acids GAMAHMGRP at the N-terminus. The 6xHis GST fragment was removed by incubation with Ni^2+^-NTA beads, and the resulting Brk1 was further purified by SEC on a superdex-75 column.

Untagged Brk1 was partially purified from 293T cells transfected with pCS2-Brk1. After 2 days of expression, cells were lysed in XB buffer (20 mM Hepes, 100 mM KCl, 1 mM MgCl_2_, 0.1 mM EDTA, pH 7.7) by nitrogen cavitation as described [11]. The extract was clarified by centrifugation (3,300 ×g, 10 min) and ultracentrifugation (45 min, 435,000 ×g; Beckman Coulter TLA120 rotor). A fraction enriched in free Brk1 was obtained by a 35%–55% ammonium sulfate cut, resuspended in XB and analyzed by SEC on a superdex-200 column run in XB on a Smart system (Amersham Pharmacia Biotech, GE Healthcare). The Brk1 containing fractions were pooled and cross-linked with 0.2% glutaraldehyde final (or several 2-fold dilutions of this maximum concentration) in the presence of 50 mM Tris, pH 8.0 for 10 min at room temperature, before immediate loading on polyacrylamide gel.

PC-Brk1 and SBP-Brk1 were purified from 293T cells transfected with appropriate pCS2 vectors. After 2 days of expression, cells were lysed in RIPA-Ca^2+^ for PC-Brk1 or RIPA for SBP-Brk1. The extract was clarified by centrifugation (3,300 ×g, 10 min) and ultracentrifugation (257,000 ×g, 1 h; Beckman Coulter 70Ti rotor). The extract was then incubated overnight with 1 ml of anti-Protein C affinity matrix for PC-Brk1 (Roche). After washing with several column volumes of RIPA-Ca^2+^, one wash in (20 mM Hepes, 100 mM KCl, 1 mM MgCl_2_, 1 mM CaCl_2_, pH 7.7), one wash with (20 mM Hepes, 100 mM KCl, 1 mM MgCl_2_, pH 7.7), PC-Brk1 was then eluted with 4 ml of (20 mM Hepes, 100 mM KCl, 5 mM EDTA, pH 7.7) during an incubation of 30 min at 4°C. For SBP-Brk1, 1 ml of ultralink streptavidin immobilized beads (Pierce) was used. After washing with several column volumes of RIPA, two washes in XB, SBP-Brk1 was eluted with 10 ml of XB supplemented with 100 µM biotin during an incubation of 30 min at room temperature. Eluted tagged Brk1 was then concentrated on Amicon Ultra filters (Millipore) down to 200 µl, and loaded on top of an 11 ml 5%–20% sucrose gradient and ultracentrifuged at 4°C (274,000 ×g, 17 h; Beckman Coulter SW41 rotor). The first top fractions containing tagged Brk1, but devoid of Wave complexes, were dialyzed twice against PBS, and flash frozen in liquid nitrogen. Tagged Brk1 was stored at −80°C until use. This procedure provides about 200 µg of PC-Brk1 and about 800 µg of SBP-Brk1 when ten 15 cm dishes of 293T cells were transfected. This procedure provided pure Brk1 in which Wave2 was not be detected by western blotting (Fig. S2).

## Results

### Brk1 is an essential subunit of the Wave complex

**Figure 1:**
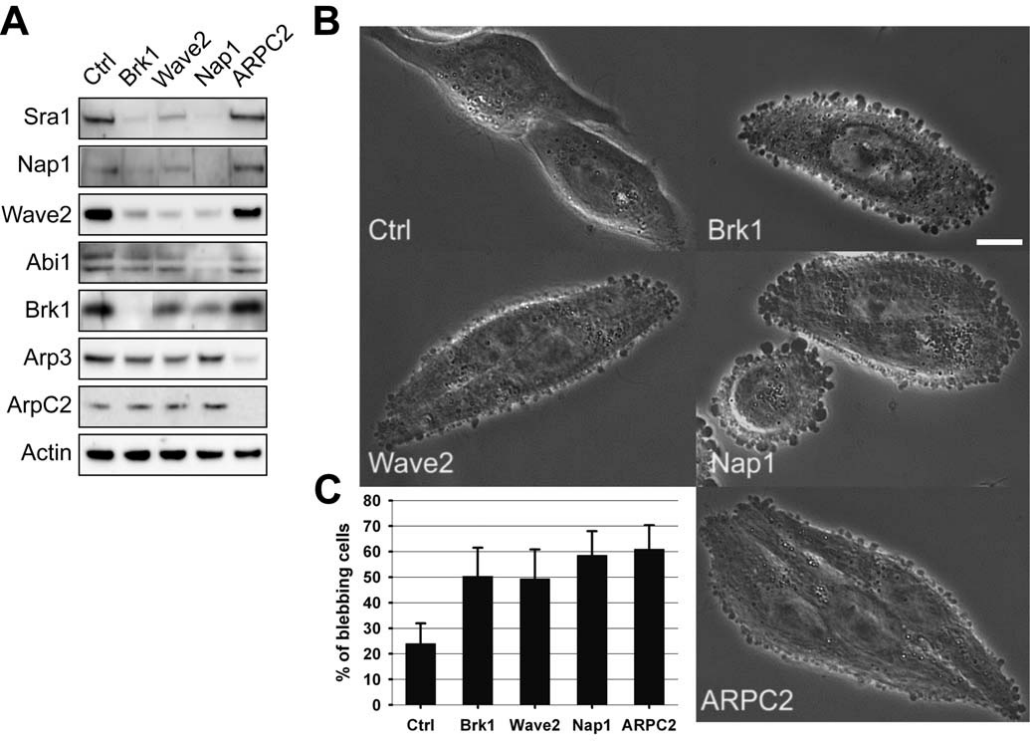
Depletion of Brk1, Wave2, Nap1 or ARPC2 induces membrane blebbing in HeLa cells. A Cells were transfected with the indicated siRNAs, cultured for three days, and cell extracts were analyzed by western blotting with the indicated antibodies. Note that non adjacent lanes from the same membrane were mounted for the sake of clarity. B Phase contrast images of living cells depleted of the Wave complex or of the Arp2/3 complex. Bar: 10 µm. C Quantification of the proportion of blebbing cells. Mean±S.D. of 5 independent experiments are plotted.

Brk1 is the least characterized subunit of the Wave complex. To evaluate the importance of Brk1 in the physiology of the complex, we knocked-down its expression in HeLa cells using siRNAs. We used a pool of 4 active siRNAs against Brk1 (Fig. S3). For comparison, we also targeted Nap1, an essential core component of the Wave complex, Wave2, the subunit activating the Arp2/3 complex [11], [14], and ARPC2, also known as p34Arc, a subunit of the Arp2/3 complex, using similar pools of siRNAs. The control population was transfected by a non-targeting pool of siRNAs. The analysis of cell lysate prepared three days after transfection of Brk1 targeting siRNAs by western blotting revealed that not only Brk1 was no longer detectable, but also that the signal of the other subunits was generally reduced (Fig. 1A). This effect was strong for Wave2, Nap1 and Sra1, slightly less pronounced for Abi1, perhaps due to other complexes containing Abi1 as previously reported [21]. Dependence of some subunits of the Wave complex on the presence of others has been previously observed in a variety of systems [13], [17], [22], [23]. The same effect was observed when Nap1 was depleted by siRNAs, except that, in this case, Abi1 level was more severely decreased. A similar effect was also obtained when Wave2 was depleted by siRNAs, even though the reduction in the signal of the other subunits was generally less pronounced than with Nap1 depletion. This might be due to the association of subunits to other Wave paralogous proteins. Upon both Nap1 and Wave2 depletion, Brk1 level was not severely affected, perhaps because Brk1 free pool was maintained. When ARPC2 was depleted, the Arp2/3 complex was also destabilized as indicated by the strong reduction in both ARPC2 and Arp3 level. However, Wave complex subunits were not affected by Arp2/3 complex depletion. When living Brk1 depleted cells were observed by phase contrast optics in the culture dish, a prominent blebbing phenotype was observed (Fig. 1B). Importantly, this blebbing phenotype was observed with all 4 siRNAs targeting Brk1 (Fig. S3). Membrane blebbing often characterized the whole periphery of Brk1 depleted cells. The same behavior was observed in Nap1, Wave2 and ARPC2 depleted cells. It should be noted that among these Brk1, Nap1, Wave2 and ARPC2 depleted cells, both round and elongated cells were found to bleb (as seen for Nap1 in Fig. 1B). Time lapse microscopy confirmed that these membrane bulges were indeed dynamic blebs, with timescales of appearance and disappearance of tens of seconds (Movie S1). Blebbing cells, defined by the presence of at least one bleb, were counted under the microscope by observing living cell culture using phase contrast optics. The proportion of blebbing Brk1-, Nap1- and ARPC2- depleted cells was significantly above the proportion of blebbing cells in the control population (Fig. 1C, P≤0.001, ANOVA followed by Holm-Sidak test of all pairwise comparisons, 5 independent experiments). The similarity of phenotypes when the Wave complex is depleted through Nap1, Wave2 or Brk1 knock-down suggests that blebbing is due to the lack of Wave complex in Brk1 depleted cells, rather than to the lack of free Brk1. The fact that Arp2/3 complex depleted cells display the same phenotype further suggests that membrane blebbing of Brk1 depleted cells is due to the reduced Wave complex activity towards the Arp2/3 complex.

Apoptosis is a well-known cause for membrane blebbing, but blebbing is also a common phenomenon during cytokinesis and cell movement (see [24] and references therein). To examine whether this blebbing phenotype was due to an induction of apoptosis, we compared Brk1 depleted cells to control cells in which apoptosis was induced by the drug staurosporine. Brk1 depleted cells were not stained with cleaved caspase3 antibody, nor did they exhibit the characteristic condensed and fragmented chromatin, suggesting that the blebbing phenotype of Brk1 depleted cells is not related to apoptosis (Fig. S4). In other words, Brk1 depleted cells that were observed to bleb on days 2, 3 and 4 after siRNA transfection are likely the same ones that bleb continuously with no incidence on their survival.

**Figure 2:**
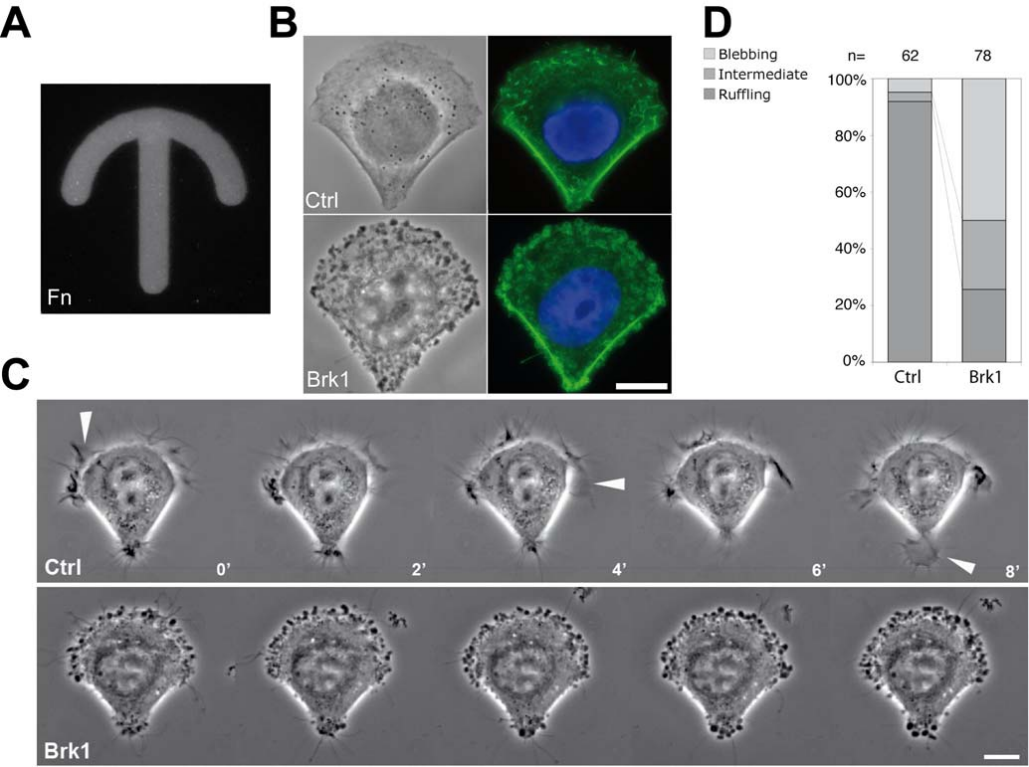
Blebbing occurs where lamellipodia and ruffles are normally emitted. A A crossbow adhesive micropattern was observed using fluorescent fibronectin (Fn). B Phase contrast (left) and fluorescence images (right) from control or Brk1 depleted cells spread on crossbow micropatterns. After fixation, filamentous actin and nuclei were stained using fluorescent phalloidin (green) and DAPI (blue), respectively. C Time-lapse microscopy of control or Brk1 depleted cells. Arrowheads indicate some lamellipodia. Time is indicated in minutes. D Multi-stage time-lapse microscopy was used to capture several cells spread on individual micro-patterns. (n) indicates the number of cells analyzed in each population. The percentage of blebbing and ruffling cells is displayed. An intermediate category was also defined for cells, which did not exhibit ruffling nor blebbing, or which exhibit both ruffles and blebs. Bars: 10 µm.

In order to characterize further the blebbing phenotype associated with Brk1 depletion, we used adhesive micropatterns in combination with time-lapse microscopy. When cells adhere on micropatterns of a crossbow shape covered with fibronectin and collagen type I (Fig. 2A), they spread and take the characteristic shape of migrating cells [19]. Both control and Brk1 depleted cells acquire this morphology, with their lateral edges contracted as evidenced by the accumulation of actin stress fibers (Fig. 2B). When cells treated with control siRNAs were recorded over several hours using phase-contrast microscopy, the large majority exhibited projections of their plasma membrane in lamellipodia or ruffles at sites where cells strongly adhere, that is primarily the large front edge and the tiny rear (Fig. 2C; Movie S2). When cells were treated with siRNAs targeting Brk1, only a minor fraction of the population exhibited lamellipodia and ruffles (Fig. 2D). Instead a large fraction exhibited membrane blebbing as previously observed in regular tissue culture dish. However in these cells, blebs were abundant at sites where membrane ruffling was observed in the control population, and scarce at the lateral edges where actin stress fibers develop. This observation further suggests that blebbing of Brk1 depleted cells occurs primarily at these sites where normally the Wave complex and the Arp2/3 complex are activated in control cells.

### The free form of Brk1 is an homotrimer

**Figure 3:**
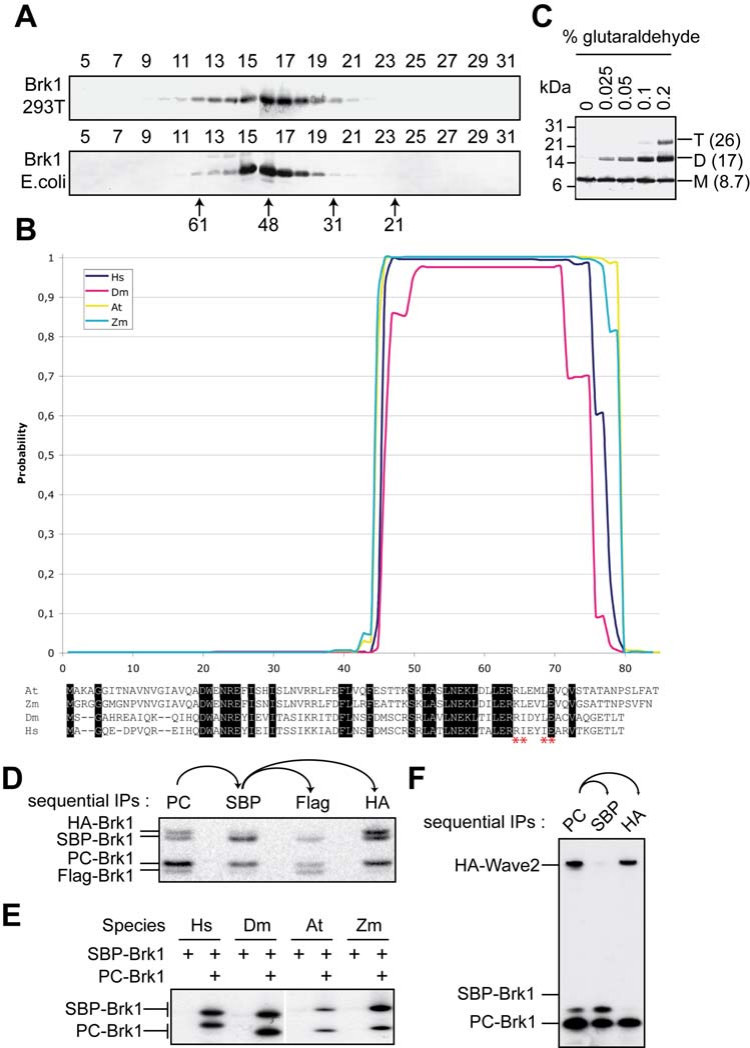
Free Brk1 is a trimer. A Partially purified untagged Brk1 expressed in 293T cells, and purified Brk1 expressed in *E.coli*, were analyzed by Size Exclusion Chromatography (SEC) using a superdex-200 column and western blotting. The numbered lanes refer to elution fractions. Stokes' radii of standards are given in Å. B Prediction of a coiled-coil in the carboxy-terminal half of Brk1 from different species (Hs: *Homo sapiens*, Dm: *Drosophila melanogaster*, At: *Arabidopsis thaliana*, Zm: *Zea mays*). In reverse lettering are indicated conserved residues in all four species. Red stars indicate the signature for trimeric coiled coils, R-h1-x-x-h2-E, where x is any amino-acid, h1 is I, L, V, or M and h2 is I, L, or V. C Free Brk1 was analyzed by western blotting after glutaraldehyde cross-linking at the indicated concentrations. The position of molecular weight markers is indicated on the left. Monomers (M), dimers (D) and trimers (T) are indicated with their calculated mass on the right. D A mixture of PC-, SBP-, Flag- and HA-Brk1 obtained by *In Vitro* Translation (IVT) was immunoprecipitated sequentially as indicated. Complexes containing at least one PC- and one SBP-Brk1 were selected by two sequential immunoprecipitations as indicated. As a third step, either Flag- or HA-Brk1 was immunoprecipitated. After three sequential immunoprecipitations, the signals for all three selected subunits are similar. This indicates that the oligomer is a trimer composed of one of each selected form of Brk1. E Oligomerization is a conserved property of Brk1. Oligomerization of Brk1 from different species (Hs: *Homo sapiens*; Dm: *Drosophila melanogaster*; At: *Arabidopsis thaliana*; Zm: *Zea mays*) was assessed by the co-translation of PC-Brk1 and SBP-Brk1 as above, and the sequential immunoprecipitations of PC-Brk1 and then of SBP-Brk1. The selection of complexes containing at least one PC- and one SBP-Brk1 in all four species indicates that Brk1 oligomerizes in all tested species, most likely trimerizes as demonstrated above for the human protein. F Wave2 binds to a single molecule of Brk1. A mixture of PC- Brk1, SBP-Brk1 and HA-Wave2 obtained by IVT was immunoprecipitated sequentially as indicated. A single PC immunoprecipitation retrieves both the trimers and Wave. SBP precipitation, as a second step, selects trimers containing both PC and SBP-Brk1, but excludes Wave2. Conversely, HA immunoprecipitation, as a second step, retrieves Wave2 bound to PC-Brk1, but excludes SBP-Brk1. Thus Brk1 does not bind to Wave2 as an oligomer.

In order to characterize the role, if any, of the free pool of Brk1, we first expanded it by transiently transfecting the untagged Brk1 cDNA in 293T cells. Brk1 overexpression did not affect the levels of Wave2 (Fig. S5). We then partially purified free Brk1. A cytosolic fraction of the transfected cells was prepared. From this cytosol, we found that free Brk1 was enriched by a 35%–55% ammonium sulfate cut that allowed us to get rid of the Wave complex, which fractionates between 20 and 30% of ammmonium sulfate (Fig. S5) [11]. This fraction enriched in free Brk1 was then analyzed by size exclusion chromatography (SEC). The Stokes' radius of Brk1 was determined to be 46 Å (Fig. 3A). For a small protein of 8.7 kDa, it was surprisingly close to the one of the aldolase marker (48 Å), which is a 160 kDa globular protein. This behavior was also displayed by recombinant Brk1 purified from *E.coli*, since this slightly longer protein ran at 50 Å (see methods). Thus, this behavior of free Brk1 is indeed due to an intrinsic property of Brk1, and does not represent another eukaryotic multiprotein complex involving Brk1. The surprising behavior of Brk1 is unlikely to result from non-specific aggregation due to excessive concentration, since purified Brk1 can be diluted 1000-fold, down to the limit of detection of western blotting, and still elutes at the same position. This behavior is strongly suggestive of an oligomeric state and/or of an elongated conformation. Along this line, a coiled coil region in the C-terminus of Brk1 from both animal and plant species is predicted with high probability by the Lupas algorithm [25]. In addition, a consensus motif that has been shown to determine the trimerization of coiled coils [26] was also conserved (Fig. 3B). The role of this motif has been first observed in the crystal structure of a 32 amino-acid long trimeric coiled coil derived from Coronin1A. This coiled coil spans 43 Å. Since this fits well with the Stokes' radius of Brk1, 46 Å, we examined whether Brk1 might indeed form a trimer.

We subjected the partially purified pool of free untagged Brk1 to glutaraldehyde cross-linking (Fig. 3C). The starting material obtained after the 35%–55% ammonium sulfate cut and SEC contains many proteins but is devoid of Wave complex (see Fig. S5). Brk1 western blot revealed new bands corresponding to a dimer and then to a trimer when glutaraldehyde was added at increasing concentrations. This result suggests that the free form of Brk1 might indeed be a trimer, but we sought to confirm this point independently of cross-linking. For this purpose, we coexpressed differently tagged Brk1 proteins and checked whether three different Brk1 variants could associate. Four forms of Brk1, tagged with Flag, PC, SBP, or HA tags at their amino-terminus, were constructed and co-produced using *in vitro* transcription and translation (IVT) in the presence of [^35^S] labeled methionine. Importantly, these four fusion proteins are resolved by SDS-PAGE and contain the same number of methionines. To analyze this complex mixture, we used sequential native immunoprecipitations, due to the property of PC and SBP precipitations to be eluted in native conditions [27]. So when the mixture of four forms was subjected to PC immunoprecipitation and eluted, complexes containing at least one PC subunit were selected. These soluble complexes were then selected for the presence of at least one SBP tagged subunit, and eluted. In the soluble complexes obtained, Flag and HA tagged Brk1 were still observed, arguing against a dimeric form (Fig. 3D). These PC and SBP selected complexes served then as the starting material for a third immunoprecipitation, either Flag or HA. When Flag-Brk1 was selected, HA-Brk1 was no longer detected and PC-, SBP- and Flag-Brk1 displayed the same intensity, indicating that trimers composed of one PC-Brk1, one SBP-Brk1 and one Flag-Brk1 were selected by these three sequential native immunoprecipitations. Conversely, when HA-Brk1 was selected, Flag-Brk1 was no longer detected and PC-, SBP-, and HA-Brk1 displayed the same intensity, indicating that trimers composed of one PC-Brk1, one SBP-Brk1 and one HA-Brk1 was selected in this case. This experiment confirms the prediction and the cross-linking result that free Brk1 is composed of trimers.

To generalize these findings to Brk1 proteins found in several species from both plant and animal kingdoms, we cloned *Drosophila*, *Arabidopsis*, and maize Brk1 in vectors permitting IVT. The Brk1 proteins were expressed by IVT in a rabbit reticulocyte lysate for the animal ones, and in the wheat germ extract for the plant ones. When PC- and SBP-Brk1 from these different species were co-translated and selected by sequential immunoprecipitations, the two proteins were specifically retrieved indicating the formation of mixed oligomers containing at least one PC- and one SBP-Brk1 (Fig. 3E). This experiment, which shows that at least dimers are formed, is compatible with trimers formed by two SBP- and one PC-Brk1, or by two PC- and one SBP-Brk1. Thus oligomerization is a conserved property of Brk1. *Drosophila* and plant Brk1 proteins are likely to form trimers as demonstrated in the case of the human Brk1 protein.

Since Brk1 is a direct partner of Wave within the Wave complex [10], [11], we next wanted to evaluate whether Brk1 trimers also binds to Wave. To this aim, we used again the sequential immunoprecipitation technique using different forms of Brk1 and co-translated *in vitro* PC-Brk1, SBP-Brk1 and HA-Wave2. After a single PC immunoprecipitation, both SBP-Brk1 and HA-Wave2 were present (Fig. 3F). Selecting trimers with SBP-Brk1 precipitation excluded Wave2, whereas selecting Wave2 with HA immunoprecipitation excluded trimers, as indicated by the loss of SBP-Brk1. This results suggests two structurally distinct conformations of Brk1, the free Brk1 trimer and the single Brk1 molecule within the Wave complex.

### Free Brk1 is a precursor in Wave complex assembly

**Figure 4:**
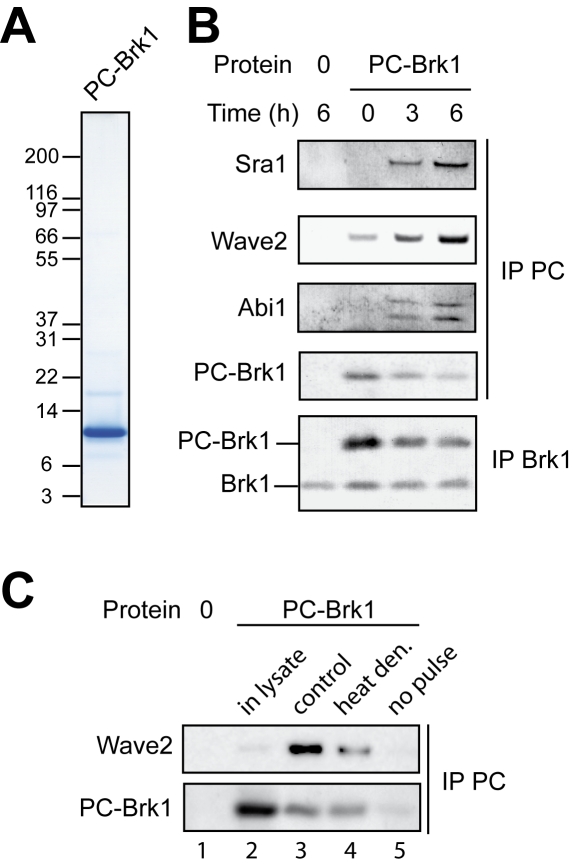
Free Brk1 incorporates into the Wave complex. A Purified PC-Brk1 was analyzed by Coomassie blue staining after SDS-PAGE. B Purified PC-Brk1 or buffer alone (0) was introduced into HeLa cells by electroporation. Cells were lysed after the indicated time. Immunoprecipitates targeting the PC tag or Brk1 were analyzed by western blotting as indicated with Sra1, Wave2, Abi1 and Brk1 antibodies. C Incorporation of exogenous Brk1 into the Wave complex was impaired compared to the control reaction (lane 3) when PC-BRK1 was added directly to the cell lysate (lane 2), and when PC-Brk1 was heat denatured (lane 4; boiled for 10 min and then cooled to room temperature before electroporation). When PC-Brk1 was put in contact to the cells, but the electroporation pulse was not delivered, PC-Brk1 does not enter cells and is washed away (lane 5).

To address the functional role of free Brk1, we first wanted to determine whether free Brk1 incorporates into the Wave complex. For this purpose, we purified free PC-Brk1 from 293T transiently transfected cells by affinity chromatography (Fig. 4A). In this preparation, PC-Brk1 molecules already incorporated into Wave complexes were eliminated by ultracentrifugation on a sucrose gradient (see Methods and Fig. S2). To examine whether free PC-Brk1 incorporates into the Wave complex, we electroporated the purified protein into HeLa cells. This procedure gives a reliable way of introducing a tuneable amount of purified protein homogenously into a cell population (Fig. S6). We introduced by electroporation a reasonable excess of PC-Brk1 over the endogenous Brk1, as judged by the Brk1 western blot of Brk1 immunoprecipitates (Fig. 4B). Exogenous PC-Brk1 decays over time. Importantly, subunits of the Wave complex were found to be associated with PC-Brk1 three hours after electroporation. The amount of associated subunits increased after six hours. Our sensitive antibody recognizing Wave2 detects some Wave2 associated with PC-Brk1 even at time 0, which in fact corresponds to about 10 minutes, the time needed to process cells from electroporation to lysis. The association of exogenous Brk1 to the Wave complex is not spontaneous and requires that Brk1 trimers enter the living cells, since addition of PC-BRK1 to cells with no electroporation pulse or the addition of PC-Brk1 to the cell lysate did not produce any incorporation of PC-Brk1 into Wave complexes (Fig. 4C). Moreover, it seems that this incorporation also required a correct trimeric conformation of Brk1 since heat denaturation of PC-Brk1 reduced its association with Wave. In an attempt to test this point further, we introduced the L57P mutation into Brk1 as a way to impair the formation of the C-terminal coiled-coil. However, this protein could not be produced and purified, suggesting that this mutation strongly destabilizes Brk1 (Fig. S7). In conclusion, this assay of Brk1 electroporation demonstrates that free Brk1 can become incorporated into the Wave complex.

To elucidate the role of this incorporation of free Brk1 into Wave complexes, we first thought that this incorporation might play a role in activating the Wave complex. Indeed, since Brk1 is a direct partner of Wave, an incorporation of Brk1 within the Wave complex would be an ideal candidate to regulate the Arp2/3 activating activity carried by Wave. This predicts that Brk1 incorporation into the Wave complex would be regulated by Rac, the small GTPase upstream of the Wave complex in the formation of lamellipodia [12], [28]. To test this prediction, we co-electroporated purified dominant-negative or active Rac proteins together with PC-Brk1. Interestingly, neither the dominant-negative RacN17, nor the active RacL61 had any effect on the level of Wave2 becoming associated to exogenous PC-Brk1, even though both proteins had a strong effect on cell spreading (Fig. S8). RacN17 prevented spreading, whereas RacL61 enhanced spreading and induced the formation of prominent lamellipodia. So this incorporation of exogenous Brk1 into the Wave complex does not appear to correlate with the activation of the Wave complex.

**Figure 5:**
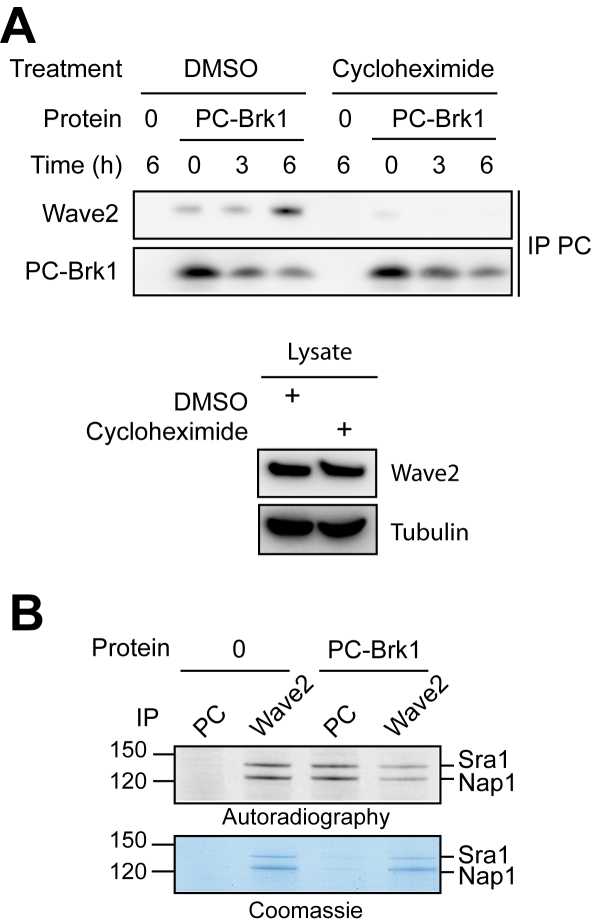
Free Brk1 incorporates into the Wave complex during its assembly. A Cycloheximide or vehicle alone (DMSO) was added to the culture medium. Inhibition of protein synthesis abolishes the association of exogenous Brk1 with Wave2, but does not affect the level of Wave2, as seen by the tubulin and Wave2 western blots in the bottom panel B Exogenous Brk1 associates preferentially with neosynthesized subunits. HeLa cells were electroporated with purified Brk1 trimers and then metabolically labeled with [^35^S] labeled methionine and cysteine. Cells were lysed and analyzed by two immunoprecipitations, the first one targeting PC analyzes what is bound to exogenous Brk1, whereas the second one targeting Wave2 in the supernatant of the first immunoprecipitation analyzes the complementary fraction of the Wave complex. After SDS-PAGE, the immunoprecipitates were stained by Coomassie blue and revealed by autoradiography. Most of the neosynthesized Sra1 and Nap1 subunits associate with the exogenous Brk1.

We then hypothesized that PC-Brk1 incorporates into the Wave complex during its assembly. This possibility was supported by a drastic block of PC-Brk1 incorporation into the Wave complex, when cycloheximide, an inhibitor of protein synthesis, was added to the culture medium 30 minutes before and after electroporation (Fig. 5A). Importantly, this is not due to an impairment of Wave2 expression, as its level was unchanged by the treatment. However, this effect of cycloheximide might be indirect. So we reasonned that if incorporation of free Brk1 occurs during the assembly of the Wave complex, then neosynthesized subunits should specifically associate with PC-Brk1. To test this possibility, cells were first electroporated with free PC-Brk1, then labeled with [^35^S] methionine and cysteine during a pulse of 15 minutes, and chased for three hours to give enough time for Wave complex assembly. After these treatments, cells were lysed and exogenous PC-Brk1 was immunoprecipitated through the PC tag. The supernatant of this immunoprecipitation was also analyzed by Wave2 immunoprecipitation, so as to examine the remaining Wave complexes containing endogenous Brk1. In the Wave2 immunoprecipitations, two bands, between 120 and 150 kDa, were visible after Coomassie staining (Fig. 5B). They were identified unambigously as the Sra1 and Nap1 subunits by western blots (Fig. S9). The bulk of the Wave complex was thus found in the second immunoprecipitation, whether or not PC-Brk1 was introduced. This argues against a rapid exchange of free Brk1 with complexed Brk1 in mature Wave complexes, otherwise most Wave complexes should be associated with PC-Brk1 that is in excess. In addition, as predicted, most of the neosynthesized subunits (about 80%), revealed by autoradiography, was associated with exogenous PC-Brk1. This experiment demonstrates that the incorporation of free Brk1 into Wave complexes occurs specifically during the assembly of the Wave complex using neosynthesized subunits.

**Figure 6:**
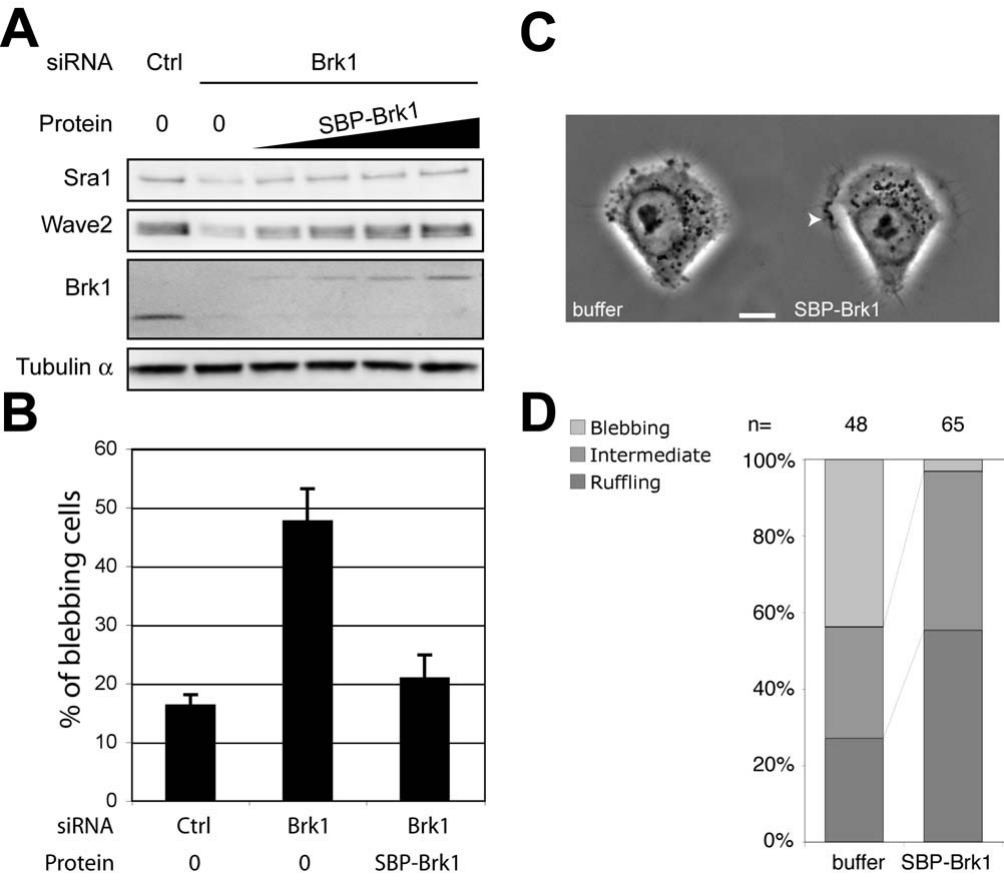
Free Brk1 is a precursor for the assembly of functional Wave complexes. HeLa cells were first depleted of Wave complexes and free Brk1 for two days using Brk1 siRNAs, then purified SBP-Brk1 was electroporated. Lysates were prepared 24 hours after electroporation and analyzed by western blotting. A Free SBP-Brk1 restores the assembly of Wave complexes in a dose-dependent manner. B Rescue of the blebbing phenotype by free Brk1. Mean±S.D. of 3 independent experiments are plotted. C Rescued cells on adhesive micropatterns also display membrane ruffles (arrowhead). D Phenotype quantification of rescued cells spread on adhesive micropatterns using multi-stage time-lapse microscopy (analysis as in figure 2D). Bar : 10 µm.

If indeed free Brk1 is used by cells to assemble Wave complexes, then introduction of these trimeric precursors should be sufficient to support Wave complex assembly in Brk1 depleted cells. HeLa cells were transfected with siRNAs targeting Brk1, and two days later electroporated with an increasing amount of purified free SBP-Brk1. One day later, cells were lysed and the amount of Wave complexes was assessed by western blotting the lysates. Indeed, increasing doses of exogenous Brk1 restored gradually the normal level of Wave complexes (Fig. 6A). This experiment establishes directly that free Brk1 is a precursor for Wave complex assembly. It should be noted that we used here SBP-Brk1 instead of PC-Brk1, because we found that SBP-Brk1 was produced and purified in greater amounts than PC-Brk1 and this experiment required greater amounts of free Brk1 than the previous ones. Nonetheless, we have checked that PC-Brk1 is also able to restore Wave complex assembly (Fig. S10). To address whether the restored Wave complexes were functional, cells were examined by phase contrast optics and the proportion of blebbing cells was determined. Restoration of Wave complex levels by SBP-Brk1 rescued the blebbing phenotype, i.e. proportion of blebbing cells was not significantly different from the control situation (Fig. 6B, P = 0.002, ANOVA followed by Holm-Sidak test of all pairwise comparisons, 3 independent experiments). A similar conclusion was reached when electroporated cells were analyzed on adhesive micropatterns by time-lapse microscopy. Not only blebbing was rescued, but also prominent ruffles were detected when Wave complexes were restored by the electroporation of SBP-Brk1 (Fig. 6C; Movie S3; Fig. 6D for a quantification).

## Discussion

### Membrane blebbing of Brk1 depleted cells

Brk1 depleted cells lacked both the free pool and the Wave complexed pool. These cells were not only affected in the formation of lamellipodia and ruffles, as expected from previous work [5], [6], [14], [15], but also they exhibited membrane blebbing. This membrane blebbing was also observed in Nap1 and Wave2 depleted cells, indicating that this defect of Brk1 depleted cells is not due to the lack of free Brk1, but rather to the lack of Wave complexes. This phenotype was previously noticed upon Sra1 or Nap1 depletion [14] and recently upon Abi1 depletion [29]. Blebbing is the likely consequence of deficient Arp2/3 mediated actin polymerization at the cell cortex, since we observed the same blebbing phenotype in Arp2/3 depleted cells. Membrane blebbing of Arp2/3 deficient cells is reported here for the first time in mammalian cells, but was recently reported in *Dictyostelium* [30]. The Wave complex is an effector of the small GTPase Rac, which controls the formation of lamellipodia [15], [28], [31]. Interestingly, the inactivation of another Rac effector, filamin, also induces prominent membrane blebbing [32]. Using adhesive micropatterns, we found that blebbing in Brk1 depleted cells occurs mostly where lamellipodia and ruffles are normally produced. This localized blebbing observed upon Brk1 depletion is in line with the recent observation that blebbing of filamin depleted cells can be abolished locally upon local perfusion of a myosin inhibitor [24]. In addition, the observation that a local release in membrane tension is a necessary and early step in the formation of lamellipodia [33] might explain the localized blebbing of Brk1 depleted cells. localized blebbing would be the result of the local decrease in membrane tension, perhaps induced by Rac activity, together with the lack of membrane projection because of Brk1 depletion. In addition, it should be mentioned that membrane blebbing has recently emerged as an alternative mechanism for cell migration [34], [35].

### In vivo assembly of the Wave complex

At the molecular level, we found that free Brk1 is an homotrimer based on SEC analysis, cross-linking and association of three different forms of Brk1. The trimeric Brk1 represents a novel complex, which seems conserved in animal and plant kingdoms. We also demonstrated here that the Brk1 trimer is an essential precursor in the assembly of Wave complexes. The evidence is three-fold: using the electroporation assay, free Brk1 was found to incorporate into the Wave complex; incorporated Brk1 specifically associates with neosynthesized subunits; and finally, Brk1 trimers are sufficient to reconstitute Wave complexes in Brk1 depleted cells. Presumably, in Brk1 depleted cells, the other subunits of the Wave complex are transcribed and translated but degraded by a quality control mechanism, because the Wave complex cannot assemble properly without Brk1. The restoration of normal levels of Wave complexes when Brk1 trimers are supplied indicates that this form comes before the assembled complex. Brk1 is the only subunit for which a free form is unambiguously detected [11]. That suggests that for the other four subunits, the synthesized proteins must incorporate rapidly into assembly intermediates or be degraded. An important question is the stoichiometry of Wave complex subunits. Indeed, using *in vitro* translation, we observed that Brk1 binds to Wave2 as a single molecule and not as a trimer. If this holds true at the level of the whole Wave complex, then it implies that the Brk1 trimer must dissociate before its incorporation into the Wave complex. Such a dissociation of the trimer requires to break the trimeric C-terminal coiled coil of Brk1. Brk1 dissociation might be spontaneous or require a post-translational modification. Further work is required to understand the molecular mechanism by which the Wave complex is assembled in cells.

The existence of this Brk1 precursor might offer a means for cells to regulate their rate of Wave complex assembly, even though in HeLa cells Brk1 trimers constitute a major pool [11], and are therefore probably not limiting the rate of Wave complex assembly. Recently, Wave2 has been found to be overexpressed in several human tumors, and this overexpression correlates with invasiveness of the tumor and poor prognostic for the patient [36]–[38]. Since Wave2 is part of a multiprotein complex, it is not sufficient to increase its gene expression to obtain an increased amount of Wave complexes. However, regulating the here described Brk1 trimeric pool or Brk1 incorporation into the Wave complex might provide a means for cells to regulate the assembly of Wave complexes.

## Supporting Information

Table S1 Conservation of Wave complex subunits.
(1.53 MB TIF)

Figure S1 Characterization of mAb 231H9 targeting Brk1. A a total cellular lysate of mouse melanoma B16F1 cells was stained by western blotting using 231H9. B B16F1 cells plated on laminin were stimulated for lamellipodia formation using AlFl3 as described (Steffen et al. 2004. Embo J 23: 749–759). Immunofluorescent staining of Brk1 decorates the tip of lamellipodia, as described for the other subunits of the Wave Complex. C 15-mer peptides covering the sequence of human Brk1 with a gliding window of 3 amino-acids were spotted on a nitrocellulose membrane (Pepspot) and reacted with 231H9. The epitope of 231H9 mAb is REIHQDWANREYIEI, a sequence perfectly conserved in human, mouse and rat Brk1.
(8.45 MB TIF)

Figure S2 Purification of free Brk1. PC-Brk1 and SBP-Brk1 preparations were all checked to be devoid of Wave complex by Wave2 western blotting. Ultracentrifugation on sucrose gradient is the step separating the Wave complex from free Brk1. The whole nitrocellulose membrane is shown after Ponceau staining to show the purification of free Brk1 along the procedure (See methods for details). 1 : lysate of 293T transfected cells 2 : lysate depleted of tagged Brk1 after the incubation with affinity beads 3 : affinity bead eluate 4 : affinity bead eluate after concentration 5 : pool of the first top fractions containing Brk1 after ultracentrifugation on sucrose gradient 6 : the same pool after concentration
(6.65 MB TIF)

Figure S3 siRNAs targeting Brk1 were tested individually or as a pool by transfecting HeLa cells. A After 3 days, cells were lysed and lysates were analyzed for their content in different Wave complex subunits by western blotting. Tubulin western blot was used as a loading control. All four siRNAs were found to be active in depleting Brk1 and Wave complexes. B All four siRNAs and the pool induced membrane blebbing when treated cells were observed by phase contrast optics in regular tissue culture dishes. Mean±S.D. of one experiment performed in duplicate. The sequences of the siRNAs are the following : #1 : sense GGGCUAACCGGGAGUACAUUU, antisense 5′-P AUGUACUCCCGGUUAGCCCUU; #2 : sense CGAUAUGUCUUGUCGUUCAUU, antisense 5′-P UGAACGACAAGACAUAUCGUU; #3 : sense ACACUAAACGAGAAAUUGAUU, antisense 5′-P UCAAUUUCUCGUUUAGUGUUU; #4 : sense GAACGGAGAAUAGAGUACAUU, antisense 5′-P UGUACUCUAUUCUCCGUUCUU.
(2.87 MB TIF)

Figure S4 Blebbing of Brk1 depleted cells is not due to apoptosis. HeLa cells transfected with control or Brk1 siRNAs were analyzed after 3 days (like in Fig. 1 and 2). Cells were fixed with 2% PFA for 15 min and permeabilized with 0.1% Triton X-100 for 5 min in PBS to preserve membrane blebs. They were then stained with cleaved Caspase 3 antibody (Rabbit mAb 5A1, Cell Signalling) and DAPI to stain chromatin. Staurosporine (STS) was used at 1 µM for 6 h as an inducer of apoptosis. Upon STS treatment, many cells became positive for cleaved caspase3 and nuclei became pyknotic, i.e. characterized by condensed and fragmented chromatin. Both of these features revealing apoptosis were absent in Brk1 depleted cells. Bar : 20 µm.
(16.20 MB TIF)

Figure S5 The partially purified free Brk1 used for crosslinking is devoid of Wave complexes. A the concentration of ammonium sulfate required to precipitate the Wave complex and free Brk1 was first evaluated in a pilot experiment. A cytosolic extract of Hela cells was subjected to 30% ammonium sulfate. The pellet was separated from the supernatant by centrifugation. The pellet obtained was resuspended in XB buffer (20 mM Hepes, 100 mM KCl, 1 mM MgCl2, 0.1 mM EDTA, pH 7.7), whereas the supernatant was adjusted to 40% ammonium sulfate. The pellet was separated from the supernatant by centrifugation. The pellet obtained was resuspended in XB buffer, whereas the supernatant was adjusted to 50% ammonium sulfate. The pellet was separated from the supernatant by centrifugation. The pellet obtained was resuspended in XB buffer, whereas the supernatant was adjusted to 60% ammonium sulfate. The pellet was separated from the supernatant by centrifugation. The pellet obtained was resuspended in XB buffer, whereas the supernatant was adjusted to 70% ammonium sulfate. The pellet was separated from the supernatant by centrifugation. The pellet obtained was resuspended in XB buffer. All the resuspended pellets were immunoprecipitated with Brk1 antibodies. The immunoprecipitates corresponding to the different ammonium sulfate pellets were Western blotted with Wave2 and Brk1 antibodies. Most Wave2 associated with Brk1, i.e. the Wave complex, precipitates with 30% ammonium sulfate as previously reported (Gautreau et al. 2004. Proc Natl Acad Sci U S A 101: 4279–4283.), whereas a significant amount of Brk1, not associated to any Wave2, was found in between 40 and 50% ammonium sulfate. B Partial purification of free Brk1 used for the crosslinking experiment displayed in figure 3C. 293T cells transfected with untagged Brk1 cDNA were lysed in XB buffer by nitrogen cavitation. The clarified lysate (see methods section) was precipitated by 35% ammonium sulfate, and the precipitate containing the Wave complex (see above panel A) was discarded.The supernatant was adjusted to 55% ammonium sulfate to precipitate free Brk1. The precipitate was resuspended in XB buffer. This constitutes the starting material (SM) for gel filtration on superdex-200 column. The elution fractions 3 to 31 were resolved by SDS-PAGE and blotted on nitrocellulose membranes. Ponceau staining and western blots using Brk1 and Wave2 antibodies are displayed. Lysates of 293T cells transfected with untagged Brk1 cDNA (L2) or empty plasmid as a control (L1). Molecular weight markers (M) were prestained (New England Biolabs). The western blots were revealed with Alkaline Phosphatase secondary antibodies using NBT/BCIP as substrates. This reaction forms a colored product directly on the nitrocellulose membrane explaining why the prestained markers are seen in the Western blots. Note that the pool of fractions containing free Brk1 used for cross-linking are devoid of Wave2 as well as the starting material obtained by the 35%–55% ammonium sulfate cut.
(4.15 MB TIF)

Figure S6 Electroporation of purified GFP into HeLa cells. The amount of electroporated GFP is indicated in µg. A GFP was immunoprecipitated after lysis of the electroporated cells. The immunoprecipitates were then analyzed by quantitative anti-GFP Western blotting, using a standard curve of the purified protein. In all conditions, about 0.1% of the protein enters the cells (Pearson correlation coefficient, R2 = 0.93). This method thus permits an immediate and tunable delivery of a protein of interest. B Immunofluorescence of GFP reveals a relatively homogenous level of GFP in electroporated cells, unlike DNA transfection. Bar : 20 µm.
(1.61 MB TIF)

Figure S7 The L57P mutation, which disrupts the predicted coiled coil, destabilizes the human Brk1 protein. A L57 is a conserved Leucine in the middle of the so-called heptad repeat motif of the C-terminal coiled coil. In reverse lettering are indicated the conserved residues highlighted in Fig. 3. Introduction of a Proline, an helix breaker residue, abolishes the prediction of a coiled coil. B HeLa cells were transfected with the empty plasmid (0), PC-Brk1 WT or L57P. Total cellular lysates were revealed with PC mAb by western blot. The position of the 6.5 kDa marker is indicated. This experiment suggests that Brk1 is strongly destabilized by a mutation that is expected to prevent its trimerization.
(10.68 MB TIF)

Figure S8 Rac activity does not modulate the incorporation of exogenous Brk1 into the Wave complexes. GST-Rac1 N17 (dominant negative) and GST-Rac1 L61 were produced and purified in E. coli as fusions with GST. GST was then cleaved off Rac1 using thrombin. Untagged Rac1 (30 µg) was mixed as indicated with PC-Brk1 (10 µg) before electroporation of HeLa cells. A Lysates of Hela cells was prepared 6 hours after electroporation. These lysates were then immunoprecipitated with PC antibody. Rac activity did not modulate the level of incorporation of PC-Brk1 into Wave complexes. The introduced Rac proteins were not detectable by western blotting the lysates, suggesting that they were introduced in little amount compared to the endogenous protein or that they were degraded during the 6 hours after electroporation. B An aliquot of above electroporated cells were spread on collagenI coated coverslips for 2 hours, then fixed and stained using fluorescent phalloidin (green) and DAPI (blue). Both RacN17 and RacL61 had clear effects. RacN17 decreased cell spreading, whereas RacL61 induced spreading by the formation of lamellipodia as detected by F-actin staining at their tip. Bar : 10 µm.
(21.20 MB TIF)

Figure S9 Whole Coomassie gel of Fig. 5A and comparison to other immunoprecipitations in order to establish that the two bands displayed in Fig.5A are indeed Sra1 and Nap1. The coomassie of Fig. 5A clearly displayed two bands in between 120 and 150 kDa in the Wave2 immunoprecipitates. These two bands were again seen in another independent Wave2 immunoprecipitation which was compared to Flag immunoprecipitations (using sepharose beads covalently coupled to the M2 mAb) of two stable lines of HEK293 cells stably transfected with an empty Flag plasmid (Flag control) or a plasmid encoding Flag tagged Abi1. As part of another project, these two bands in the Flag-Abi1 immunoprecipitate were identified by Mass Spectrometry to be Sra1 and Nap1 (from top to bottom, respectively, as indicated). This point is here further validated by Sra1 and Nap1 western blotting of these three immunoprecipitates.
(3.60 MB TIF)

Figure S10 Trimeric PC-Brk1 restores the assembly of Wave complexes. HeLa cells were first depleted of Wave complexes using Brk1 siRNAs for two days, and free PC-Brk1 or buffer alone (0) was then electroporated. Lysates were prepared 24 hours after electroporation and analyzed by western blotting with the indicated antibodies.
(0.66 MB TIF)

Movie S1 Bleb dynamics. HeLa cells transfected with non-targeting siRNAs (Ctrl) or siRNAs targeting Brk1, Wave2, Nap1 or ARPC2 were recorded by time-lapse microscopy using phase contrast optics. Cells were seeded on glass coverslips, 24 hrs earlier. Fig. 1B is extracted from this movie. Bar : 10 µm.
(4.81 MB MOV)

Movie S2 Localized blebbing. HeLa cells transfected with non-targeting (Ctrl) or Brk1 targeting siRNAs were adhered on crossbow adhesive micropatterns and recorded by time-lapse microscopy using phase contrast optics. Hour:min are indicated. Fig. 2C is derived from this movie. Bar : 10 µm.
(3.00 MB MOV)

Movie S3 Rescue of the blebbing phenotype of Brk1 depleted cells by electroporation of free SBP-Brk1. HeLa cells were transfected with Brk1 targeting siRNAs, and two days later were electroporated with buffer, or SBP-Brk1. One day later, cells were spread on crossbow adhesive micropatterns and recorded by time-lapse microscopy using phase contrast optics. Time is indicated in minutes. Bar : 10 µm.
(5.08 MB MOV)
