# Supplementary figures and images for: Free Brick1 Is a Trimeric Precursor in the Assembly of a Functional Wave Complex
Source: PLoS One. 2008 Jun 18;3(6):e2462. doi: 10.1371/journal.pone.0002462 (PMC2413427; doi:10.1371/journal.pone.0002462)

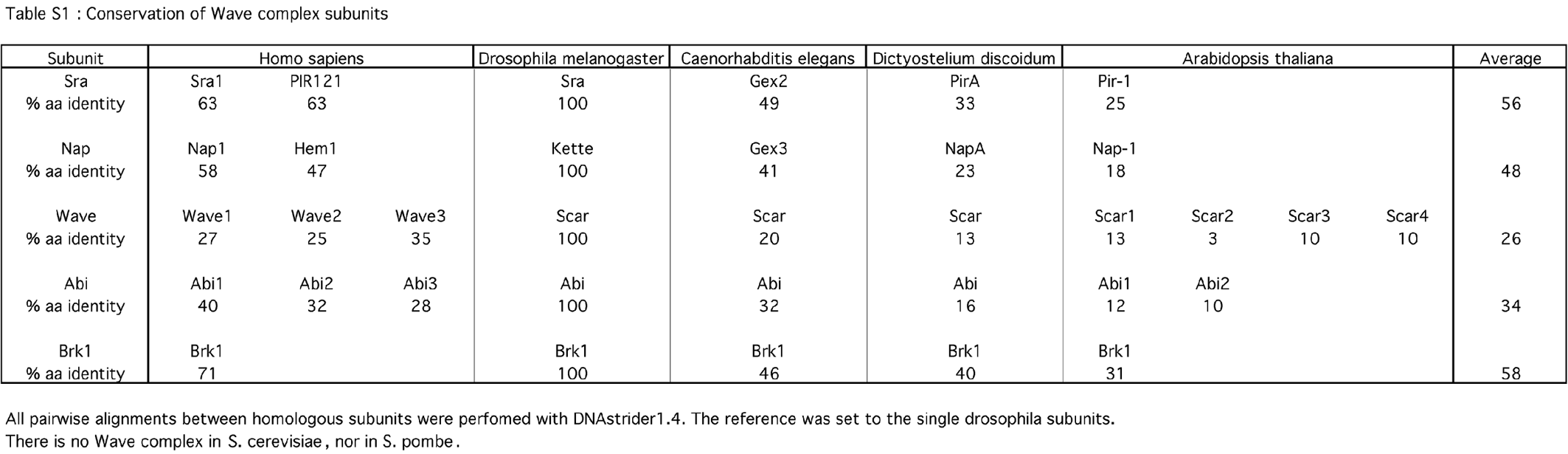

Supplement: Table S1 — Conservation of Wave complex subunits. (1.53 MB TIF) [file pone.0002462.s001.tif]

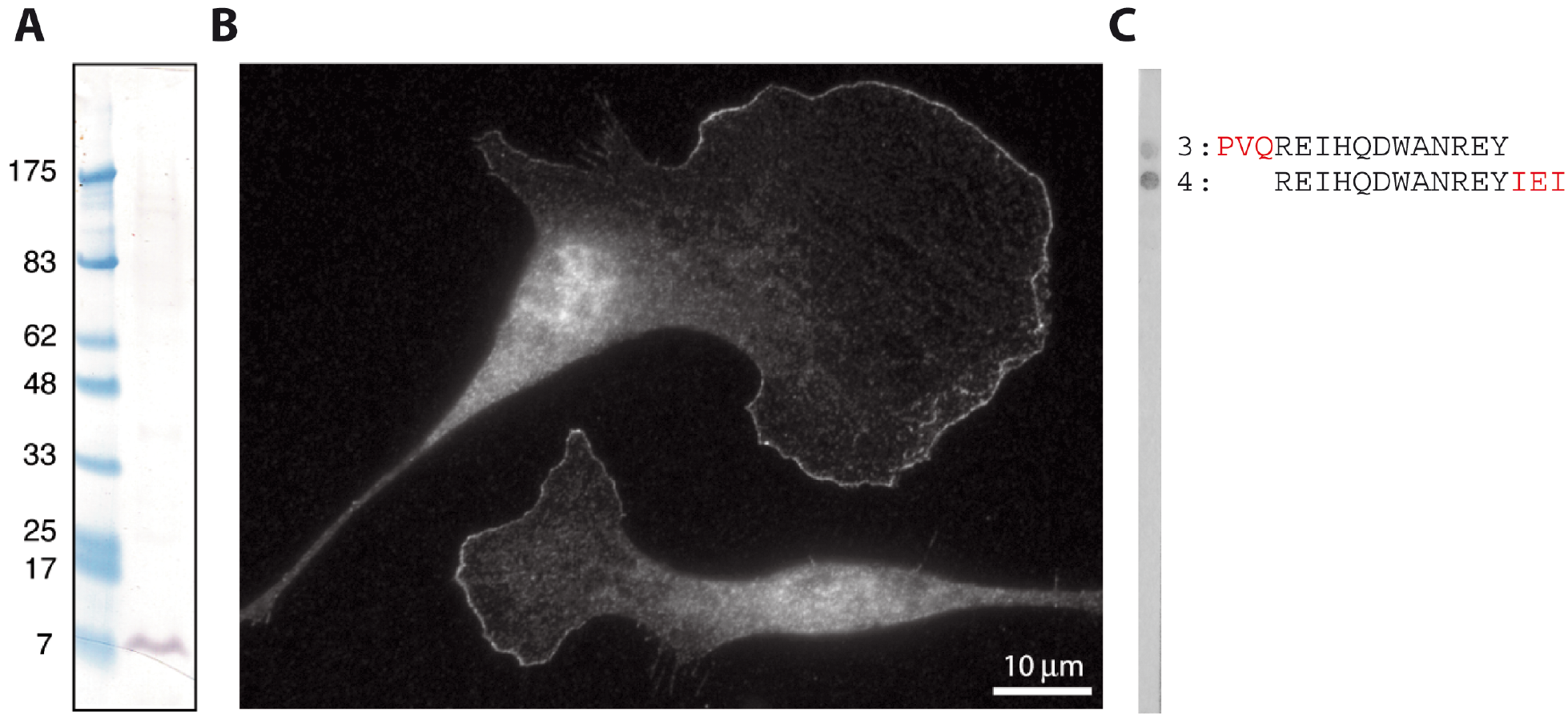

Supplement: Figure S1 — Characterization of mAb 231H9 targeting Brk1. A a total cellular lysate of mouse melanoma B16F1 cells was stained by western blotting using 231H9. B B16F1 cells plated on laminin were stimulated for lamellipodia formation using AlFl3 as described (Steffen et al. 2004. Embo J 23: 749–759). Immunofluorescent staining of Brk1 decorates the tip of lamellipodia, as described for the other subunits of the Wave Complex. C 15-mer peptides covering the sequence of human Brk1 with a gliding window of 3 amino-acids were spotted on a nitrocellulose membrane (Pepspot) and reacted with 231H9. The epitope of 231H9 mAb is REIHQDWANREYIEI, a sequence perfectly conserved in human, mouse and rat Brk1. (8.45 MB TIF) [file pone.0002462.s002.tif]

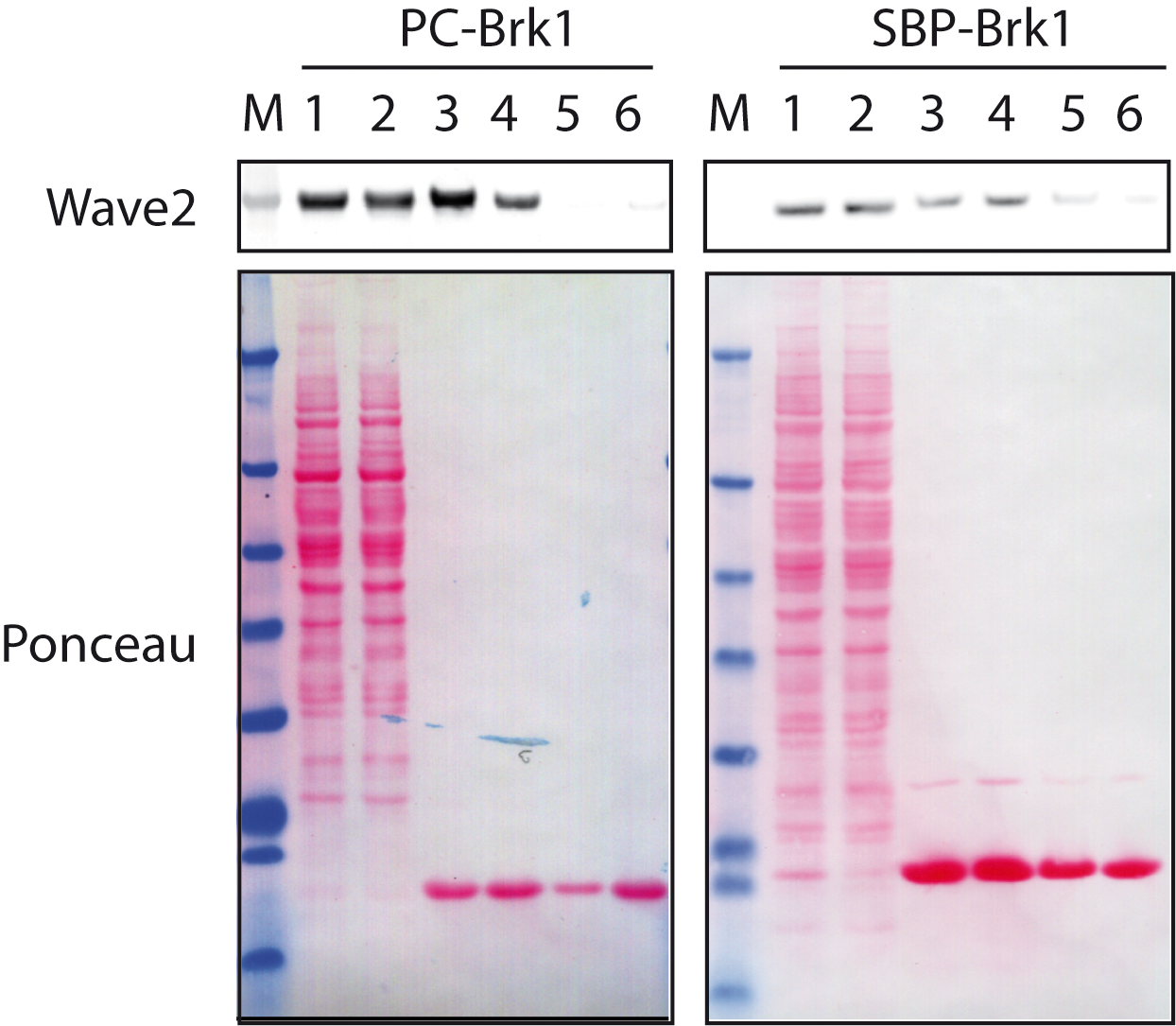

Supplement: Figure S2 — Purification of free Brk1. PC-Brk1 and SBP-Brk1 preparations were all checked to be devoid of Wave complex by Wave2 western blotting. Ultracentrifugation on sucrose gradient is the step separating the Wave complex from free Brk1. The whole nitrocellulose membrane is shown after Ponceau staining to show the purification of free Brk1 along the procedure (See methods for details). 1 : lysate of 293T transfected cells 2 : lysate depleted of tagged Brk1 after the incubation with affinity beads 3 : affinity bead eluate 4 : affinity bead eluate after concentration 5 : pool of the first top fractions containing Brk1 after ultracentrifugation on sucrose gradient 6 : the same pool after concentration (6.65 MB TIF) [file pone.0002462.s003.tif]

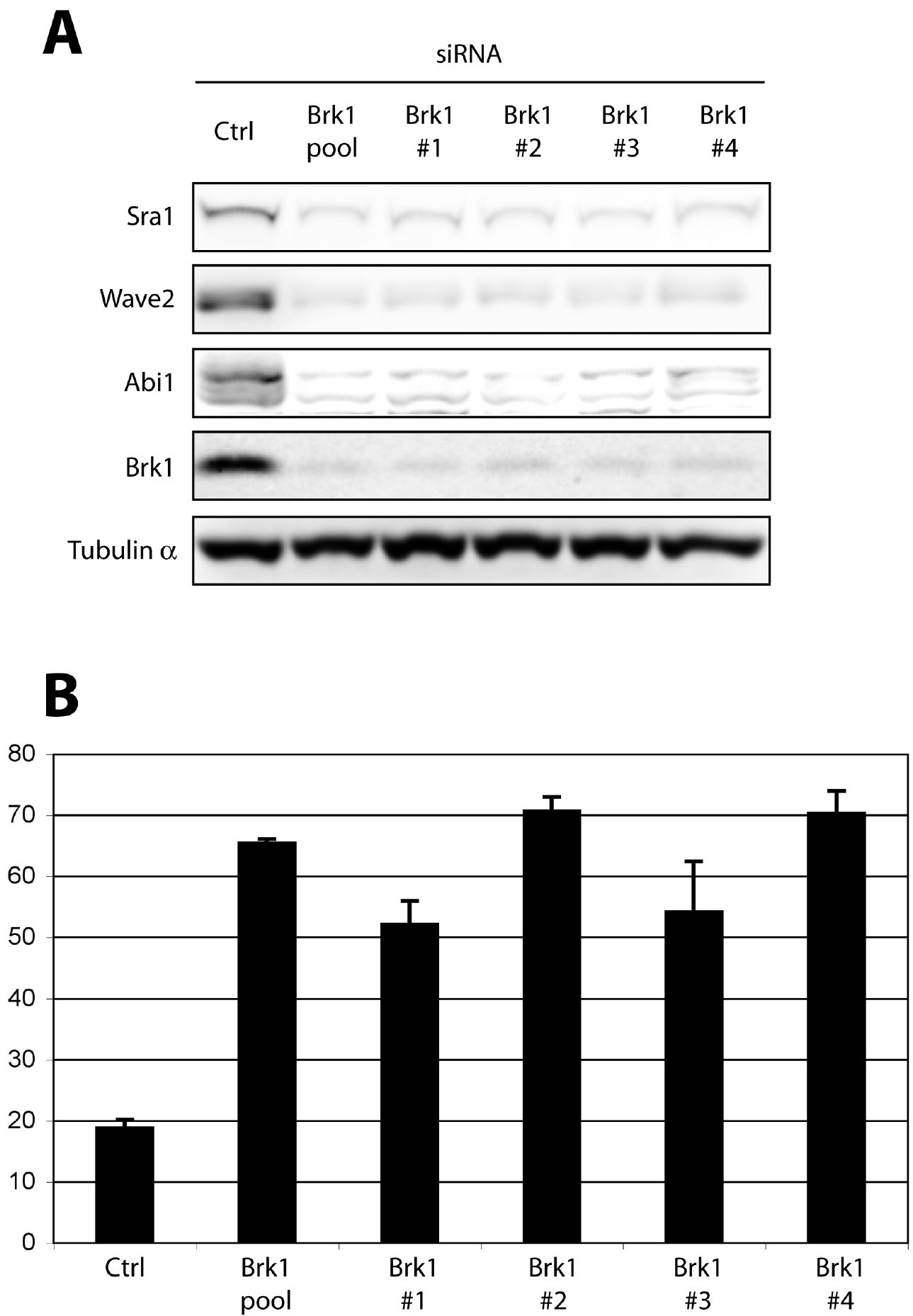

Supplement: Figure S3 — siRNAs targeting Brk1 were tested individually or as a pool by transfecting HeLa cells. A After 3 days, cells were lysed and lysates were analyzed for their content in different Wave complex subunits by western blotting. Tubulin western blot was used as a loading control. All four siRNAs were found to be active in depleting Brk1 and Wave complexes. B All four siRNAs and the pool induced membrane blebbing when treated cells were observed by phase contrast optics in regular tissue culture dishes. Mean±S.D. of one experiment performed in duplicate. The sequences of the siRNAs are the following : #1 : sense GGGCUAACCGGGAGUACAUUU, antisense 5′-P AUGUACUCCCGGUUAGCCCUU; #2 : sense CGAUAUGUCUUGUCGUUCAUU, antisense 5′-P UGAACGACAAGACAUAUCGUU; #3 : sense ACACUAAACGAGAAAUUGAUU, antisense 5′-P UCAAUUUCUCGUUUAGUGUUU; #4 : sense GAACGGAGAAUAGAGUACAUU, antisense 5′-P UGUACUCUAUUCUCCGUUCUU. (2.87 MB TIF) [file pone.0002462.s004.tif]

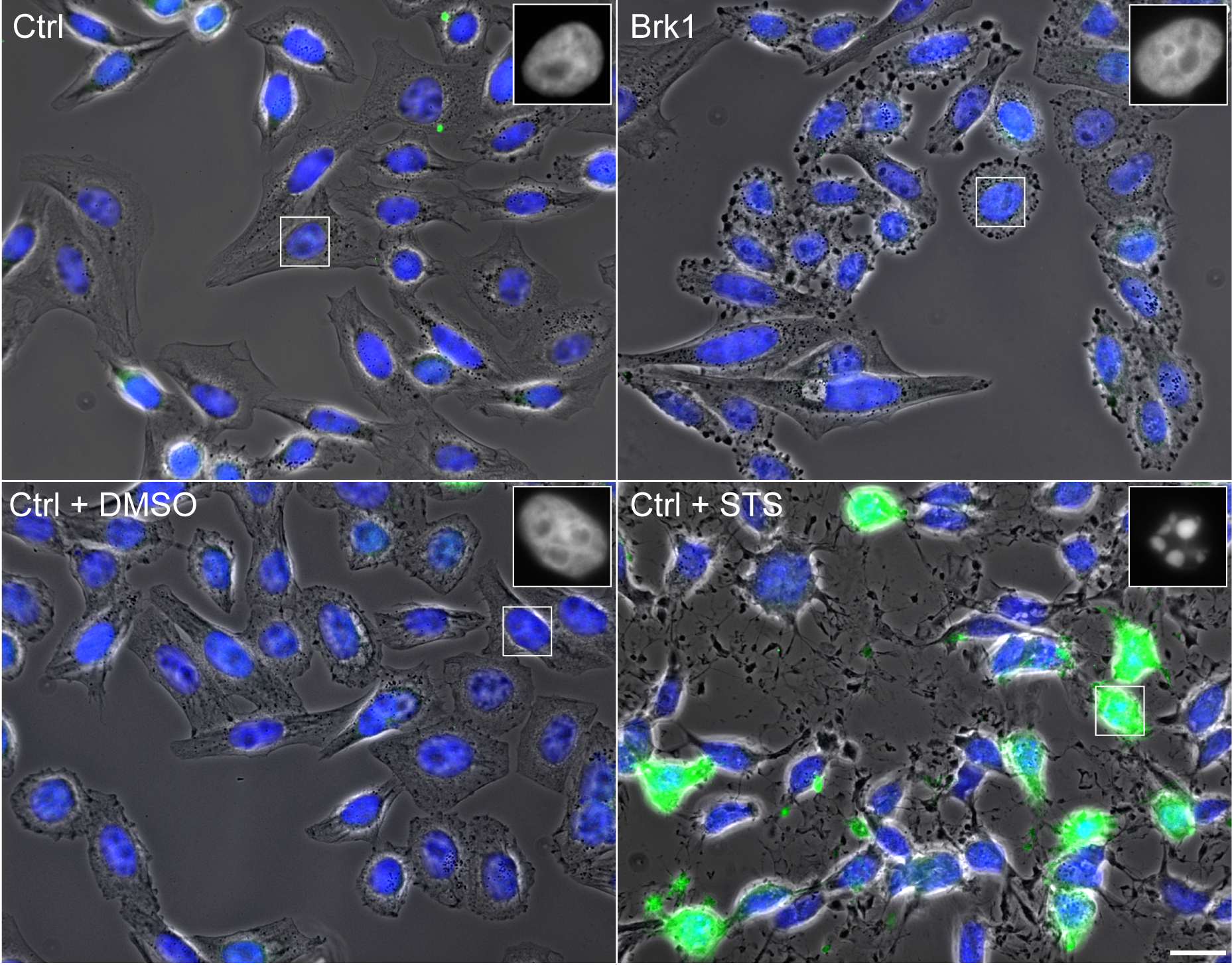

Supplement: Figure S4 — Blebbing of Brk1 depleted cells is not due to apoptosis. HeLa cells transfected with control or Brk1 siRNAs were analyzed after 3 days (like in Fig. 1 and 2). Cells were fixed with 2% PFA for 15 min and permeabilized with 0.1% Triton X-100 for 5 min in PBS to preserve membrane blebs. They were then stained with cleaved Caspase 3 antibody (Rabbit mAb 5A1, Cell Signalling) and DAPI to stain chromatin. Staurosporine (STS) was used at 1 µM for 6 h as an inducer of apoptosis. Upon STS treatment, many cells became positive for cleaved caspase3 and nuclei became pyknotic, i.e. characterized by condensed and fragmented chromatin. Both of these features revealing apoptosis were absent in Brk1 depleted cells. Bar : 20 µm. (16.20 MB TIF) [file pone.0002462.s005.tif]

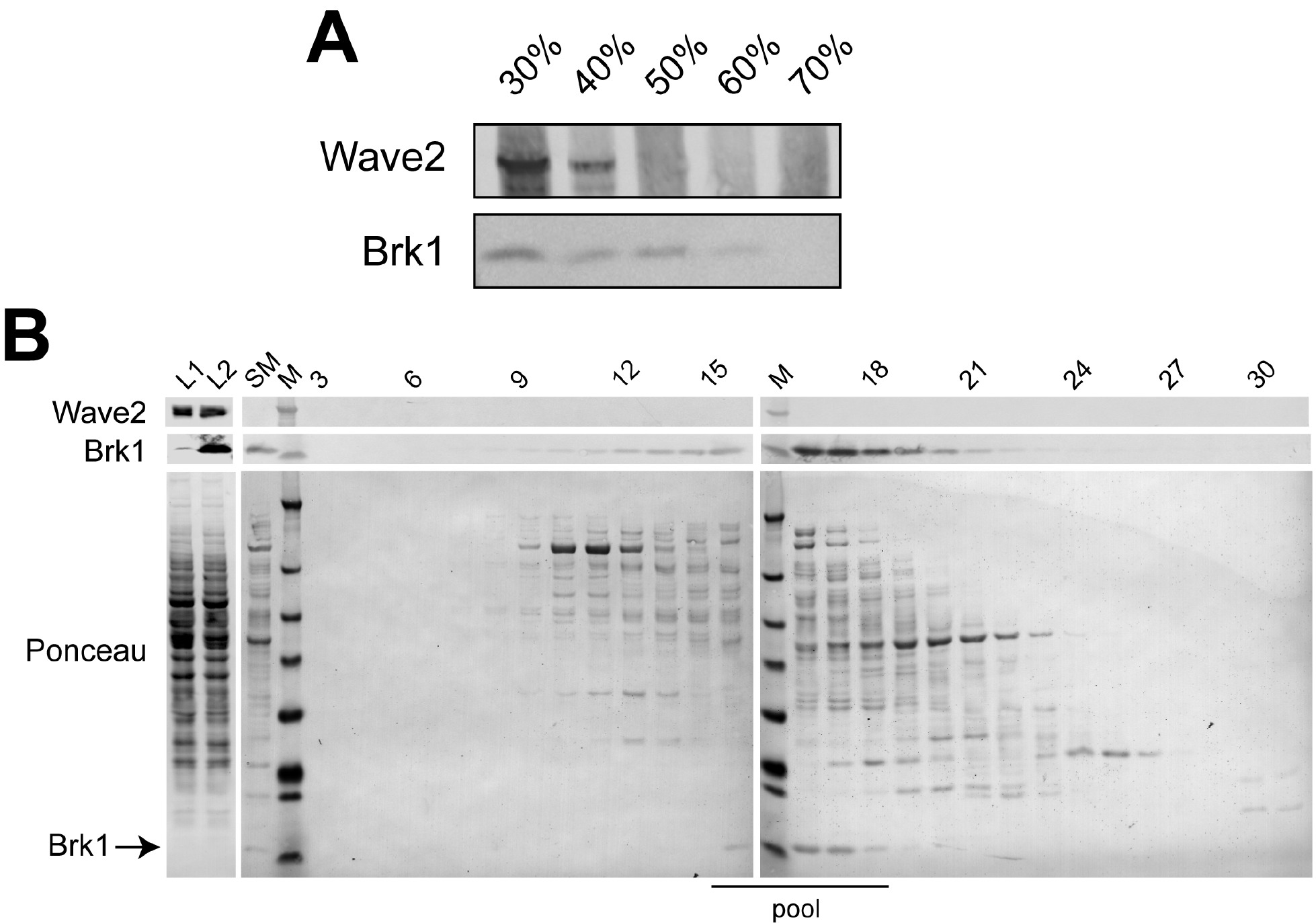

Supplement: Figure S5 — The partially purified free Brk1 used for crosslinking is devoid of Wave complexes. A the concentration of ammonium sulfate required to precipitate the Wave complex and free Brk1 was first evaluated in a pilot experiment. A cytosolic extract of Hela cells was subjected to 30% ammonium sulfate. The pellet was separated from the supernatant by centrifugation. The pellet obtained was resuspended in XB buffer (20 mM Hepes, 100 mM KCl, 1 mM MgCl2, 0.1 mM EDTA, pH 7.7), whereas the supernatant was adjusted to 40% ammonium sulfate. The pellet was separated from the supernatant by centrifugation. The pellet obtained was resuspended in XB buffer, whereas the supernatant was adjusted to 50% ammonium sulfate. The pellet was separated from the supernatant by centrifugation. The pellet obtained was resuspended in XB buffer, whereas the supernatant was adjusted to 60% ammonium sulfate. The pellet was separated from the supernatant by centrifugation. The pellet obtained was resuspended in XB buffer, whereas the supernatant was adjusted to 70% ammonium sulfate. The pellet was separated from the supernatant by centrifugation. The pellet obtained was resuspended in XB buffer. All the resuspended pellets were immunoprecipitated with Brk1 antibodies. The immunoprecipitates corresponding to the different ammonium sulfate pellets were Western blotted with Wave2 and Brk1 antibodies. Most Wave2 associated with Brk1, i.e. the Wave complex, precipitates with 30% ammonium sulfate as previously reported (Gautreau et al. 2004. Proc Natl Acad Sci U S A 101: 4279–4283.), whereas a significant amount of Brk1, not associated to any Wave2, was found in between 40 and 50% ammonium sulfate. B Partial purification of free Brk1 used for the crosslinking experiment displayed in figure 3C. 293T cells transfected with untagged Brk1 cDNA were lysed in XB buffer by nitrogen cavitation. The clarified lysate (see methods section) was precipitated by 35% ammonium sulfate, and the precipitate containing the Wave [file pone.0002462.s006.tif]

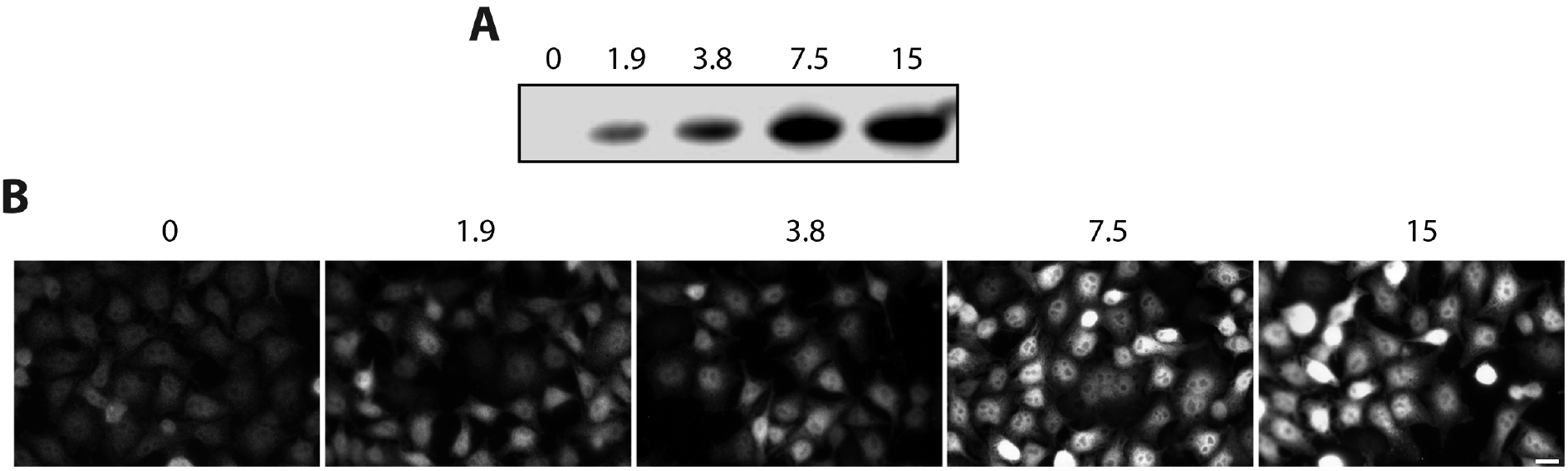

Supplement: Figure S6 — Electroporation of purified GFP into HeLa cells. The amount of electroporated GFP is indicated in µg. A GFP was immunoprecipitated after lysis of the electroporated cells. The immunoprecipitates were then analyzed by quantitative anti-GFP Western blotting, using a standard curve of the purified protein. In all conditions, about 0.1% of the protein enters the cells (Pearson correlation coefficient, R2 = 0.93). This method thus permits an immediate and tunable delivery of a protein of interest. B Immunofluorescence of GFP reveals a relatively homogenous level of GFP in electroporated cells, unlike DNA transfection. Bar : 20 µm. (1.61 MB TIF) [file pone.0002462.s007.tif]

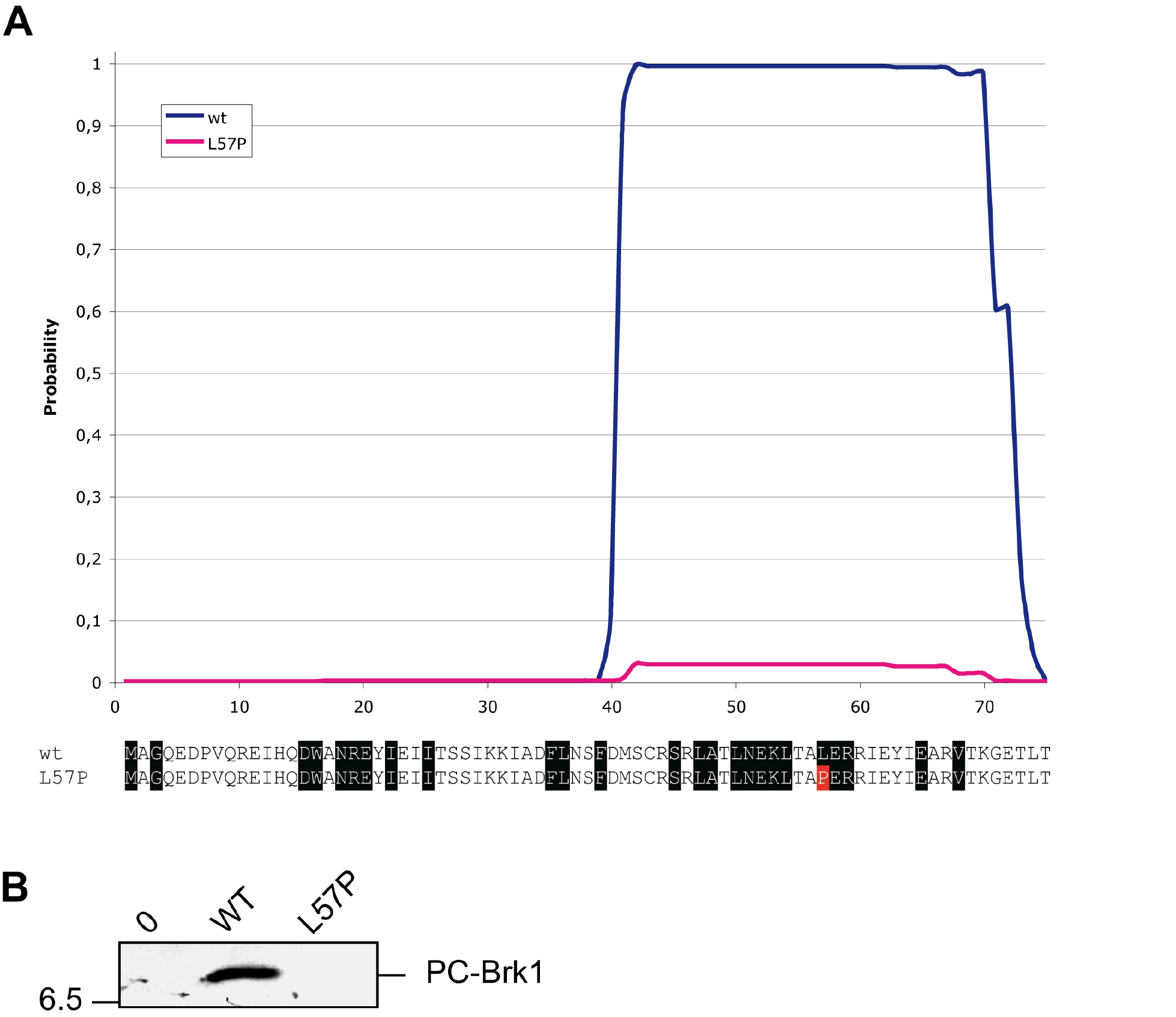

Supplement: Figure S7 — The L57P mutation, which disrupts the predicted coiled coil, destabilizes the human Brk1 protein. A L57 is a conserved Leucine in the middle of the so-called heptad repeat motif of the C-terminal coiled coil. In reverse lettering are indicated the conserved residues highlighted in Fig. 3. Introduction of a Proline, an helix breaker residue, abolishes the prediction of a coiled coil. B HeLa cells were transfected with the empty plasmid (0), PC-Brk1 WT or L57P. Total cellular lysates were revealed with PC mAb by western blot. The position of the 6.5 kDa marker is indicated. This experiment suggests that Brk1 is strongly destabilized by a mutation that is expected to prevent its trimerization. (10.68 MB TIF) [file pone.0002462.s008.tif]

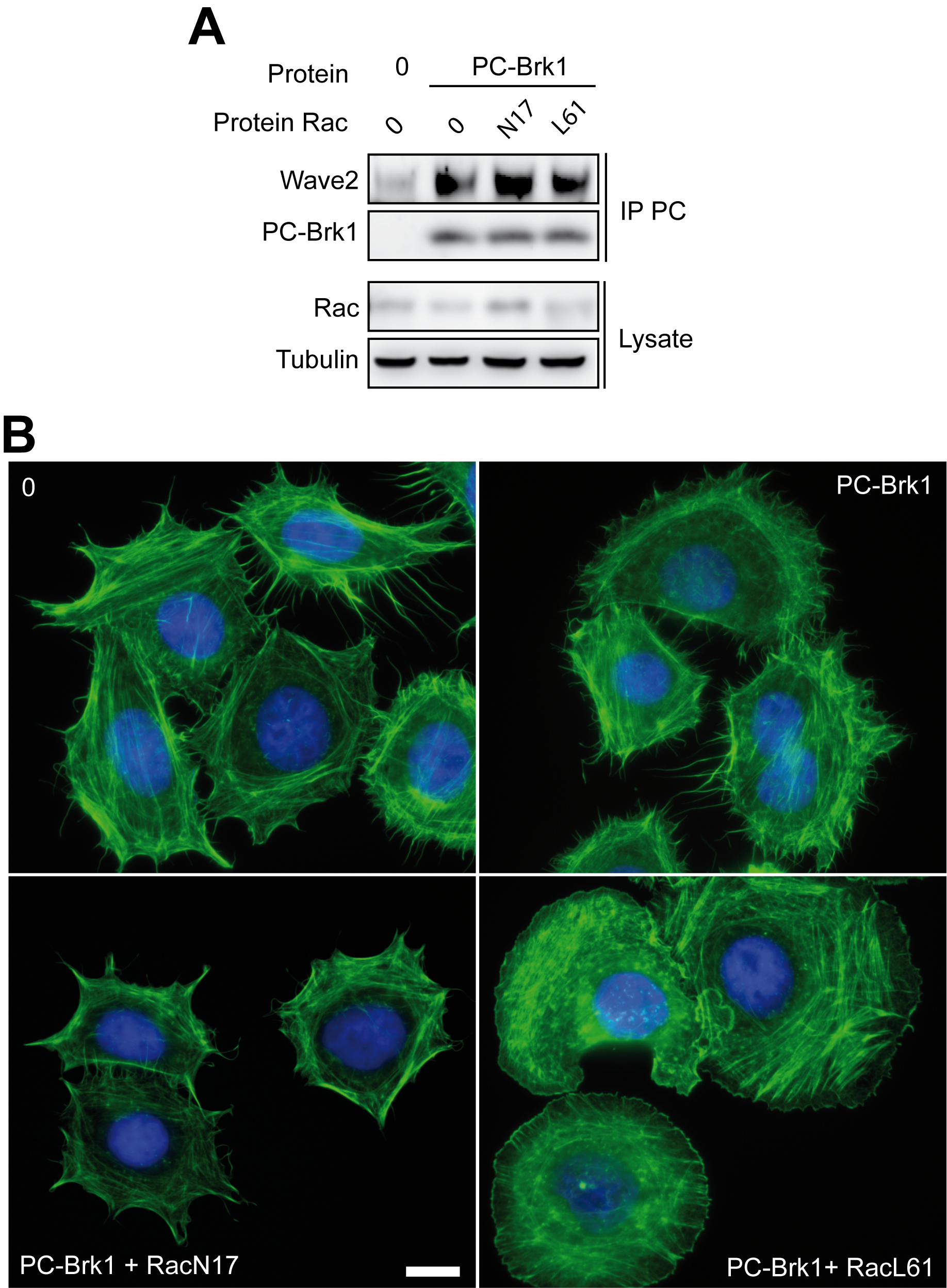

Supplement: Figure S8 — Rac activity does not modulate the incorporation of exogenous Brk1 into the Wave complexes. GST-Rac1 N17 (dominant negative) and GST-Rac1 L61 were produced and purified in E. coli as fusions with GST. GST was then cleaved off Rac1 using thrombin. Untagged Rac1 (30 µg) was mixed as indicated with PC-Brk1 (10 µg) before electroporation of HeLa cells. A Lysates of Hela cells was prepared 6 hours after electroporation. These lysates were then immunoprecipitated with PC antibody. Rac activity did not modulate the level of incorporation of PC-Brk1 into Wave complexes. The introduced Rac proteins were not detectable by western blotting the lysates, suggesting that they were introduced in little amount compared to the endogenous protein or that they were degraded during the 6 hours after electroporation. B An aliquot of above electroporated cells were spread on collagenI coated coverslips for 2 hours, then fixed and stained using fluorescent phalloidin (green) and DAPI (blue). Both RacN17 and RacL61 had clear effects. RacN17 decreased cell spreading, whereas RacL61 induced spreading by the formation of lamellipodia as detected by F-actin staining at their tip. Bar : 10 µm. (21.20 MB TIF) [file pone.0002462.s009.tif]

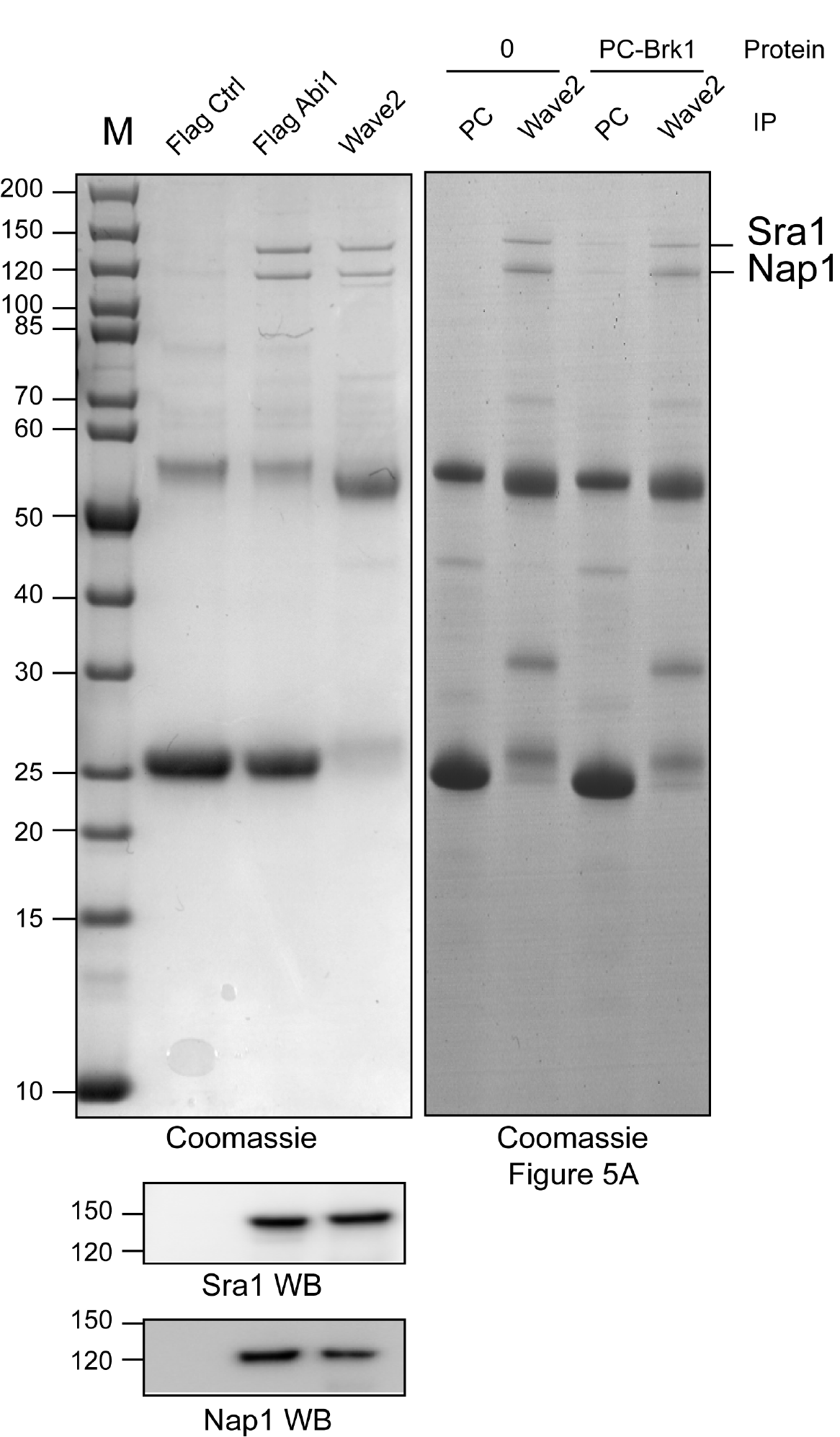

Supplement: Figure S9 — Whole Coomassie gel of Fig. 5A and comparison to other immunoprecipitations in order to establish that the two bands displayed in Fig.5A are indeed Sra1 and Nap1. The coomassie of Fig. 5A clearly displayed two bands in between 120 and 150 kDa in the Wave2 immunoprecipitates. These two bands were again seen in another independent Wave2 immunoprecipitation which was compared to Flag immunoprecipitations (using sepharose beads covalently coupled to the M2 mAb) of two stable lines of HEK293 cells stably transfected with an empty Flag plasmid (Flag control) or a plasmid encoding Flag tagged Abi1. As part of another project, these two bands in the Flag-Abi1 immunoprecipitate were identified by Mass Spectrometry to be Sra1 and Nap1 (from top to bottom, respectively, as indicated). This point is here further validated by Sra1 and Nap1 western blotting of these three immunoprecipitates. (3.60 MB TIF) [file pone.0002462.s010.tif]

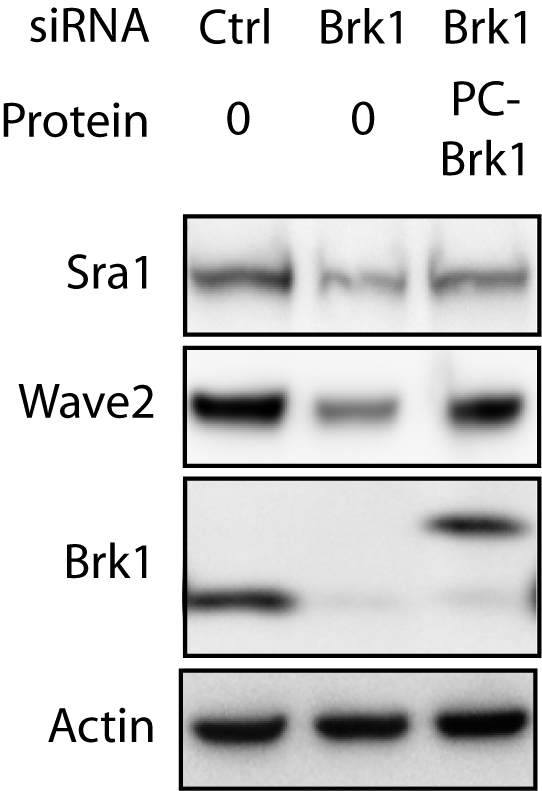

Supplement: Figure S10 — Trimeric PC-Brk1 restores the assembly of Wave complexes. HeLa cells were first depleted of Wave complexes using Brk1 siRNAs for two days, and free PC-Brk1 or buffer alone (0) was then electroporated. Lysates were prepared 24 hours after electroporation and analyzed by western blotting with the indicated antibodies. (0.66 MB TIF) [file pone.0002462.s011.tif]
